# Hypertrophic cardiomyopathy in *MYBPC3* carriers in aging

**DOI:** 10.20517/jca.2023.29

**Published:** 2024-01-11

**Authors:** Kalyani Ananthamohan, Julian E. Stelzer, Sakthivel Sadayappan

**Affiliations:** 1 Department of Internal Medicine, Division of Cardiovascular Health and Disease, University of Cincinnati, Cincinnati, OH 45267, USA.; 2 Department of Physiology and Biophysics, School of Medicine, Case Western Reserve University, Cleveland, OH 45267, USA.

**Keywords:** Age-related HCM, *MYBPC3*, alternative splicing, nonsense-mediated decay, ubiquitin-proteosome system, chaperone-mediated autophagy

## Abstract

Hypertrophic cardiomyopathy (HCM) is characterized by abnormal thickening of the myocardium, leading to arrhythmias, heart failure, and elevated risk of sudden cardiac death, particularly among the young. This inherited disease is predominantly caused by mutations in sarcomeric genes, among which those in the cardiac myosin binding protein-C3 (*MYBPC3*) gene are major contributors. HCM associated with *MYBPC3* mutations usually presents in the elderly and ranges from asymptomatic to symptomatic forms, affecting numerous cardiac functions and presenting significant health risks with a spectrum of clinical manifestations. Regulation of *MYBPC3* expression involves various transcriptional and translational mechanisms, yet the destiny of mutant *MYBPC3* mRNA and protein in late-onset HCM remains unclear. Pathogenesis related to *MYBPC3* mutations includes nonsense-mediated decay, alternative splicing, and ubiquitin-proteasome system events, leading to allelic imbalance and haploinsufficiency. Aging further exacerbates the severity of HCM in carriers of *MYBPC3* mutations. Advancements in high-throughput omics techniques have identified crucial molecular events and regulatory disruptions in cardiomyocytes expressing *MYBPC3* variants. This review assesses the pathogenic mechanisms that promote late-onset HCM through the lens of transcriptional, post-transcriptional, and post-translational modulation of *MYBPC3*, underscoring its significance in HCM across carriers. The review also evaluates the influence of aging on these processes and *MYBPC3* levels during HCM pathogenesis in the elderly. While pinpointing targets for novel medical interventions to conserve cardiac function remains challenging, the emergence of personalized omics offers promising avenues for future HCM treatments, particularly for late-onset cases.

## Graphical Abstract

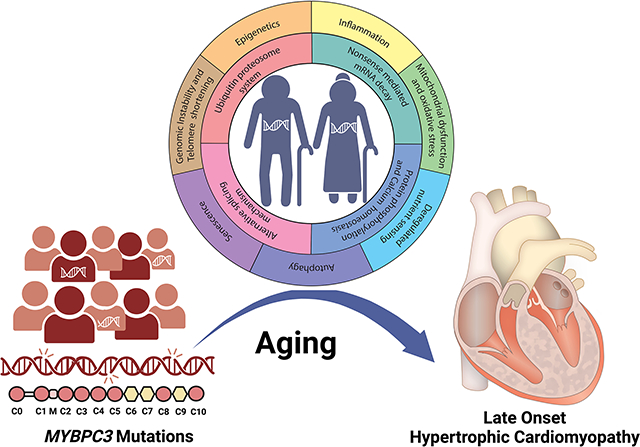

## INTRODUCTION

Hypertrophic Cardiomyopathy (HCM) impacts as many as 1 in every 200 people^[1]^, affecting approximately 36 million people worldwide^[2,3]^. HCM is characterized by thickening of the heart muscle, with or without left ventricular outflow obstruction, and is associated with diastolic dysfunction^[4,5]^. Sudden cardiac death (SCD) is strongly linked to HCM, particularly in young adults and highly trained elite athletes^[6]^. HCM is a primary genetic cause of pathological left ventricular hypertrophy and heart failure, a significant indicator of cardiac-related morbidity and mortality^[7–9]^. Typically, treatments for HCM are pharmacologic drug administration to treat symptoms and, in some cases, myectomy to prevent progression to heart failure (HF)^[10,11]^. Treating the precise fundamental defects of sarcomere function is critical to further progress in preventing HCM complications. HCM is caused predominantly by inherited mutations in the sarcomeric proteins, and it is the resultant contractile abnormalities that impair cardiac function^[12]^. Over 50%-60% of all inherited HCMs^[13,14]^ are caused by mutations in the *MYBPC3* gene that encodes the cardiac isoform of the sarcomeric protein cardiac myosin-binding protein-C (cMyBP-C). cMyBP-C is critical for both normal systolic contraction and complete relaxation in diastole^[15–21]^. However, the precise molecular mechanisms at play in *MYBPC3* mutations, including those that result in impaired muscle function leading to HCM, SCD, and HF, remain to be elucidated.

HCM has been considered a heterogeneous cardiac disease with incomplete penetrance and its variable phenotypic presentation, clinical manifestation, genetic etiology, age of onset, and severity. As they age, carriers are at considerable risk of developing significant clinical symptoms. Among all genes, mutations in *MYBPC3* are associated with late-onset HCM^[22]^. The first clinical manifestation seen in carriers of *MYBPC3* mutations is a mild to moderate phenotype^[23–26]^. Therefore, it is essential to determine the molecular mechanisms that underlie pathogenic phenotype in late onset and aging. Accordingly, this review aims to shed light on the contribution of age to HCM phenotype and examine the molecular mechanisms that drive age-dependent HCM penetrance in *MYBPC3* gene carriers.

## PHYSIOLOGY, PATHOPHYSIOLOGY, AND CLINICAL MANIFESTATIONS OF HCM, A DISEASE OF THE SARCOMERE

### HCM phenotype

**Figure 1.**
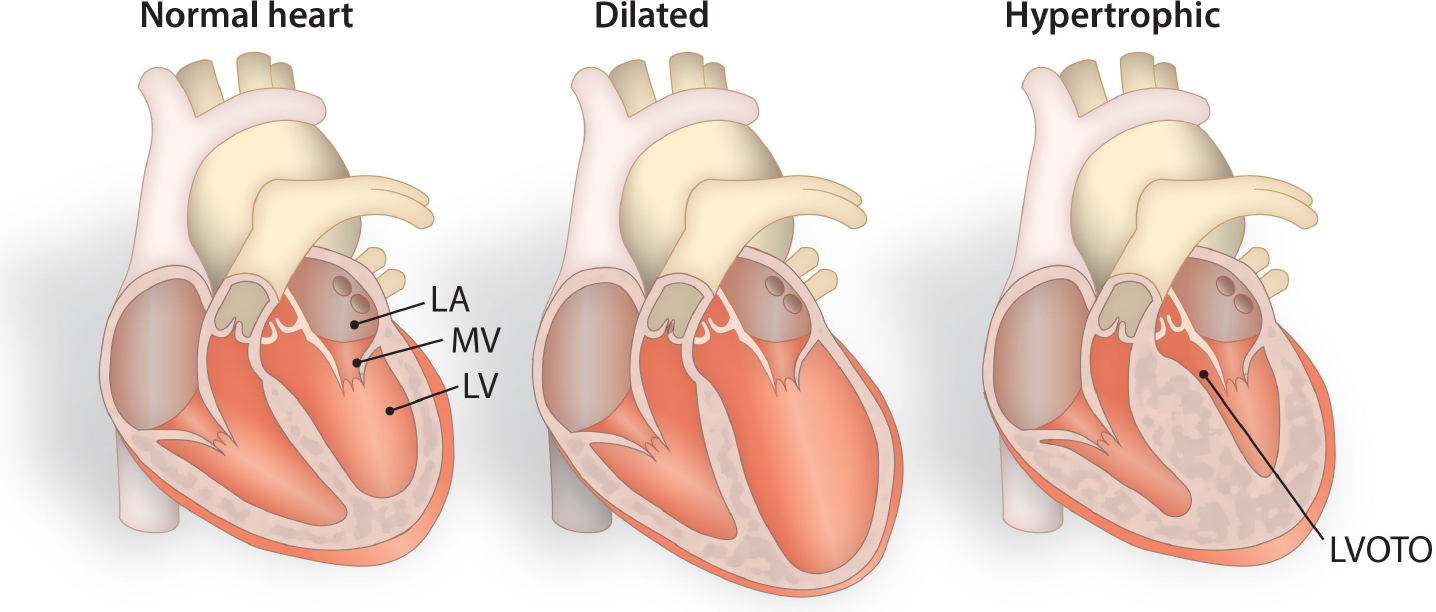
Schematic diagram differentiating normal hearts from dilated and hypertrophied hearts. Cardiomyopathies occur due to genetic variations, resulting in distinct physiological and/or pathophysiological consequences. In terms of clinical manifestations, cardiomyocytes within hypertrophied hearts become enlarged and demonstrate cardiac dysfunction due to increased left ventricular wall thickness, diminished left ventricular cavity size, and altered blood flow rates. LA-Left Atrium, MV-Mitral Valve, LV-Left Ventricle, LVOTO-Left Ventricular Outflow Tract Obstruction.

HCM is characterized by thickening of the heart muscle, with or without left ventricular outflow obstruction, in association with diastolic dysfunction [Figure 1]^[5]^. Sudden cardiac death is strongly linked to HCM, particularly in young adults and trained athletes^[6]^. Clinically, HCM is defined by increased left ventricular wall thickness (end-diastolic left ventricular wall thickness ≥ 15mm) or the equivalent relative to the body surface area in children^[4,23,25]^. Diminished LV wall thickness (13–14 mm) is also considered a diagnostic feature if the patient is either genetically test-positive or has a family history of HCM^[27,28]^. Hypertrophied myocardial fibers with disordered muscle bundles and interstitial fibrosis are additional typical diagnostic features. Additionally, luminal narrowing owing to high wall thickness in coronary microvasculature, myocardial ischemia injury, and fibrosis are observed^[5,27,29]^. Diverse phenotypic expression and naturally variable progression of HCM are reflections of a range of clinical manifestations from dyspnea and/or syncope to sudden cardiac death. A subset of HCM also evolves into restrictive cardiomyopathy (RCM) or dilated cardiomyopathy (DCM) [Figure 1]. Based on its clinical features, HCM disease progression can be classified into subclinical HCM, classical HCM, adverse remodeling, and end-stage HCM or overt HCM with left ventricular ejection fraction (LVEF) falling below 50%^[11,27]^. Most HCM cases exhibit a genetic mutation in one or multiple of the sarcomeric genes.

### HCM is a disease of the sarcomeric genes

**Figure 2.**
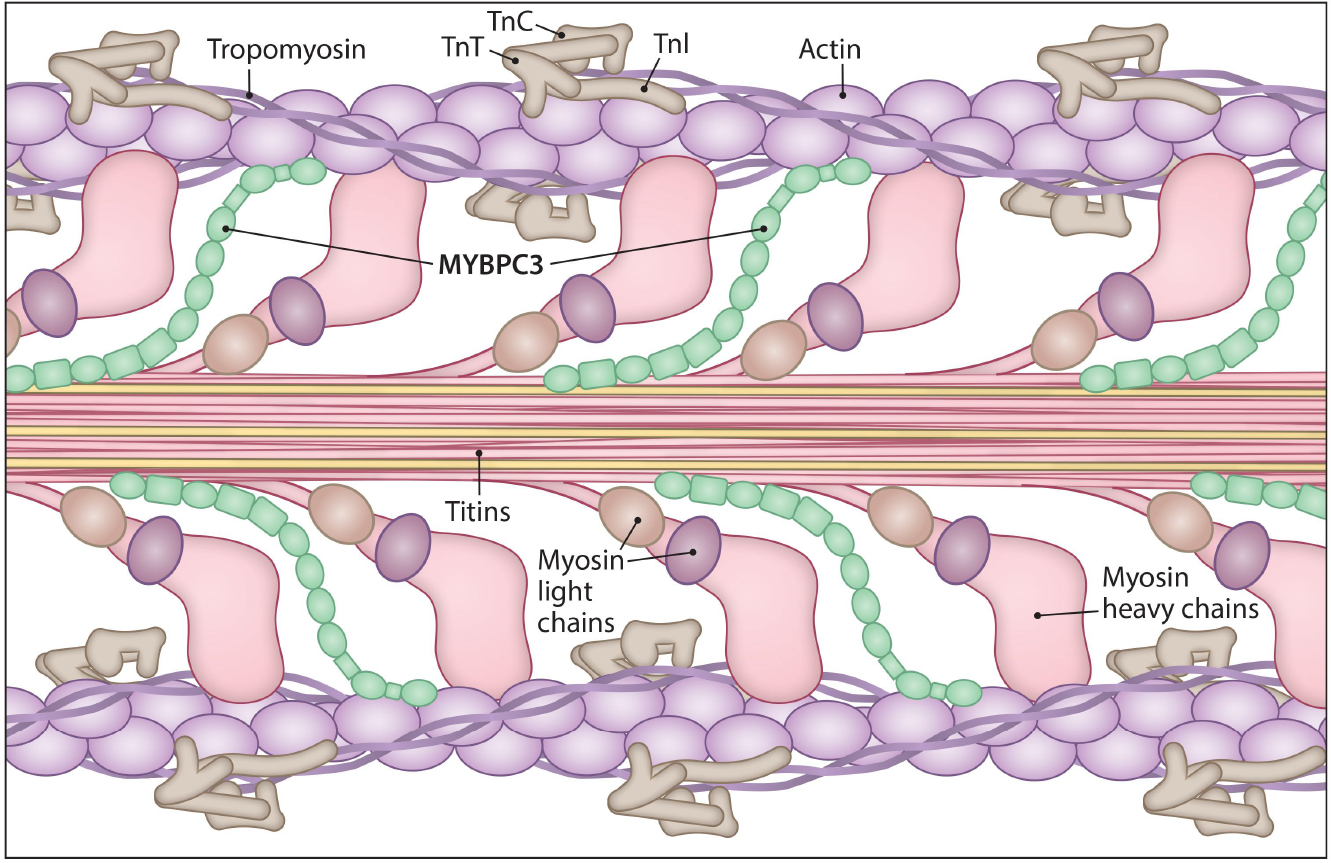
Structure and arrangements of myofilament protein in C-zone of cardiac sarcomere. The myofilaments within the sarcomere consist of two types: thick and thin filaments. The thick filaments are primarily composed of myosin protein. Each myosin molecule consists of a heavy chain that forms a tail and terminates with a globular head. Additionally, light chains interact with these globular heads. The giant protein titin spans across the sarcomere and is surrounded by myosin. The thin filaments are constituted by actin, forming a helical structure that interacts with the myosin globular heads. Cardiac myosin binding protein-C (*MYBPC3*), the cardiac paralog, is a regulatory protein that associates with actin and myosin, binding at its N-terminal and interacting with titin at its C-terminal. The thin filament protein, tropomyosin, wraps around the actin helix, and the three complex subunits of other thin filament proteins, known as cardiac troponins, namely Troponin C (TnC), Troponin T (TnT), and Troponin I (TnI), are also present.

Extensive research has been conducted on gene mutations causing HCM. A primary focus of these studies is *MYH7*, a myosin heavy chain gene a major component of the thick filaments of the sarcomere. Further studies have been conducted on other sarcomeric genes, including *MYBPC3*, cardiac troponin T (*TNNT2*), tropomyosin 1 (*TPM1*), myosin light chain 2 (*MYL2*), myosin light chain (*MYL3*), troponin I (*TNNI3*), and α-actinin (*ACTC1*) [Figure 2]^[30]^. Mutations in *MYH7* and *MYBPC3* genes are, however, the most frequent causes of HCM. Along with these sarcomeric proteins, regulatory proteins, such as calcium-handling proteins, are essential in maintaining sarcomere functions, but they also comprise a genetic subset of HCM disease phenotypes^[31]^. About 30%-60% of HCM individuals are estimated to harbor sarcomere gene mutations^[32,33]^. However, the expression of disease typically arises from a complex interplay of multiple mutations or a blend of causal genes and other factors, such as regulatory genes, epigenetics, and environmental influences^[31]^. Similarly, pathogenic sarcomere variants exacerbate existing conditions, such as ventricular arrhythmia, HF, atrial fibrillation (AF), stroke, or even death in HCM individuals^[32,34]^. Among the 50 unique cardiomyopathy genes with truncating mutations, *MYBPC3*, *TNNT2*, and phospholamban (*PLN*) exhibited a high ratio of cases compared to reference populations for HCM^[35,36]^. Genetic mutations altering cardiac function are mostly missense mutations and truncating mutations. However, *MYBPC3* truncating mutations (e.g., nonsense, frameshift, ± 1,2 splice) are predominant among HCM probands^[35–38]^.

## AGING AS A RISK FACTOR FOR HCM

Phenotypic expression of HCM is age-dependent^[39]^ and presents unique clinical features with various causative genes and phenotypic variations from mild to severe symptoms^[40]^. HCM-related crude mortality rates (CMR) stratified by age groups during the study period from 1999 to 2019 demonstrate that HCM mortality rates among U.S. residents were highest among those aged 75 and above. The crude mortality rate decreased for other age groups (15–29 years, 30–44 years, 45–59 years) from 2010 to 2019. However, no significant change was reported in the CMR of HCM patients aged 60 to 74 and over 75^[41]^. A follow-up study from the Sarcomeric Human Cardiomyopathy Registry observed higher mortality rates owing to HCM compared to the general U.S. population of similar age^[34]^. However, this mortality rate was still highest for those 60 to 69 years of age. Otherwise, the risk of adverse events, such as ventricular arrhythmia, HF, AF, stroke, or death, was highest for individuals diagnosed with HCM before the age of 40, suggesting a milder effect of these events in the elderly compared to younger individuals^[32,34]^. However, the left ventricle (LV) end-diastolic diameter and atrial diameter are larger in the elderly with more mitral valve calcification. Although SCD is common among the young or adolescents, clinical presentation of LV remodeling, LV dilation, and HF are more common in midlife and beyond, statistically strengthening the existing assertion that age is a strong influencing factor for HCM presentation^[31]^. Apart from age, incomplete penetrance is also influenced by sex since women have a greater propensity to develop late-onset HCM^[41–43]^, and previous reports have shown that females with a pronounced HCM presentation had a poor prognosis^[44]^. Although estrogen has cardioprotective effects, women may be more susceptible to diastolic dysfunction, which is the earliest sign of HCM^[45]^. Reducing levels of estrogen during the transition phase of menopause might be a plausible explanation for the increased risk of women developing HCM 6 to 13 years later in life compared to men^[46]^.

In addition to clinical features, genetic screening for the sarcomeric gene was likely to be negative in elderly HCM patients^[47]^. In a large cohort of 488 HCM probands, DNA was analyzed for mutations in the protein-coding exons of 8 common sarcomere genes (*MYBPC3, MYH7, MYL2, MYL3*, *TNNT2, TNNI3, TPM1*, and *ACTC1*)^[47]^. Only 22% of HCM patients diagnosed after the age of 45 years had an identifiable mutation, whereas 49% of patients diagnosed before the age of 45 years had a mutation in the same set of genes^[31,48]^. Thus, these population studies seem to forecast the aging process as a major influencing factor for HCM, irrespective of genotype. At the same time, early genetic testing allows more treatment options for genotype-positive individuals. The clinical features observed in genotype-positive and -negative individuals are heterogeneous, but age is a common contributor to the late onset of cardiac dysfunction. To account for and understand the molecular events underlying the etiology of HCM, we hereinafter only consider the key biochemical cascades triggered by either gene mutation or age. Systematic reviews published on the genetic basis and the role of aging in HCM are excellent resources for gaining further perspectives^[49,50]^.

### Molecular determinants of HCM

In HCM, cardiomyocyte hypertrophy is a major histological feature that results from a variety of pathways, including stress-sensing signaling pathways, the expression of trophic and mitotic genes, such as TGFβ1 and its downstream targets, the classical mitogen-activated protein kinase (MAPK) pathway, the phosphoinositide 3-kinase (PI3K) pathway, and the calcineurin pathway^[49]^. However, in addition to genetic mutations, environmental and epigenetic factors also act as upstream modulators of these signal transduction pathways to initiate hypertrophy- related events^[51]^. Epigenetic factors are inheritable changes that cause gene silencing by DNA methylation, histone deacetylation, and microRNAs (miRNAs)^[51–53]^. Another crucial epigenetic mechanism, histone modification, has also been suspected of posing a risk for cardiovascular diseases. For example, histone acetyltransferase (HAT) p300 is upregulated in heart diseases^[54]^, and inhibition of histone deacetylase 2 (HDAC2) induces a hypertrophic signal^[55]^. The contribution of DNA methylation and its associated molecules in the hypertrophic phenotype of cardiomyocytes is still undetermined. In the past few decades, post-transcriptional gene silencing of cardiac proteins by miRNAs has been extensively explored and found to be associated with myocardial infarction, arrhythmogenic cardiomyopathy, Long QT syndrome, and cardiomyopathies. The role of miRNAs in cardiac diseases and cardiac hypertrophy is not within the scope of the current review and miRNAs with presumed significance in cardiomyopathies are listed in [Table 1].

### Molecular pathways in aging and the cardiac aging process

In most cases, the cause of HCM is inherited, while age-dependent penetrance is influenced by the variability in cellular responses to specific signals or stimuli. As just discussed, epigenetic factors are key effectors of phenotype in genetic diseases. The influence of aging on these epigenetic factors is addressed next in this review.

#### Aging/senescence

**Figure 3.**
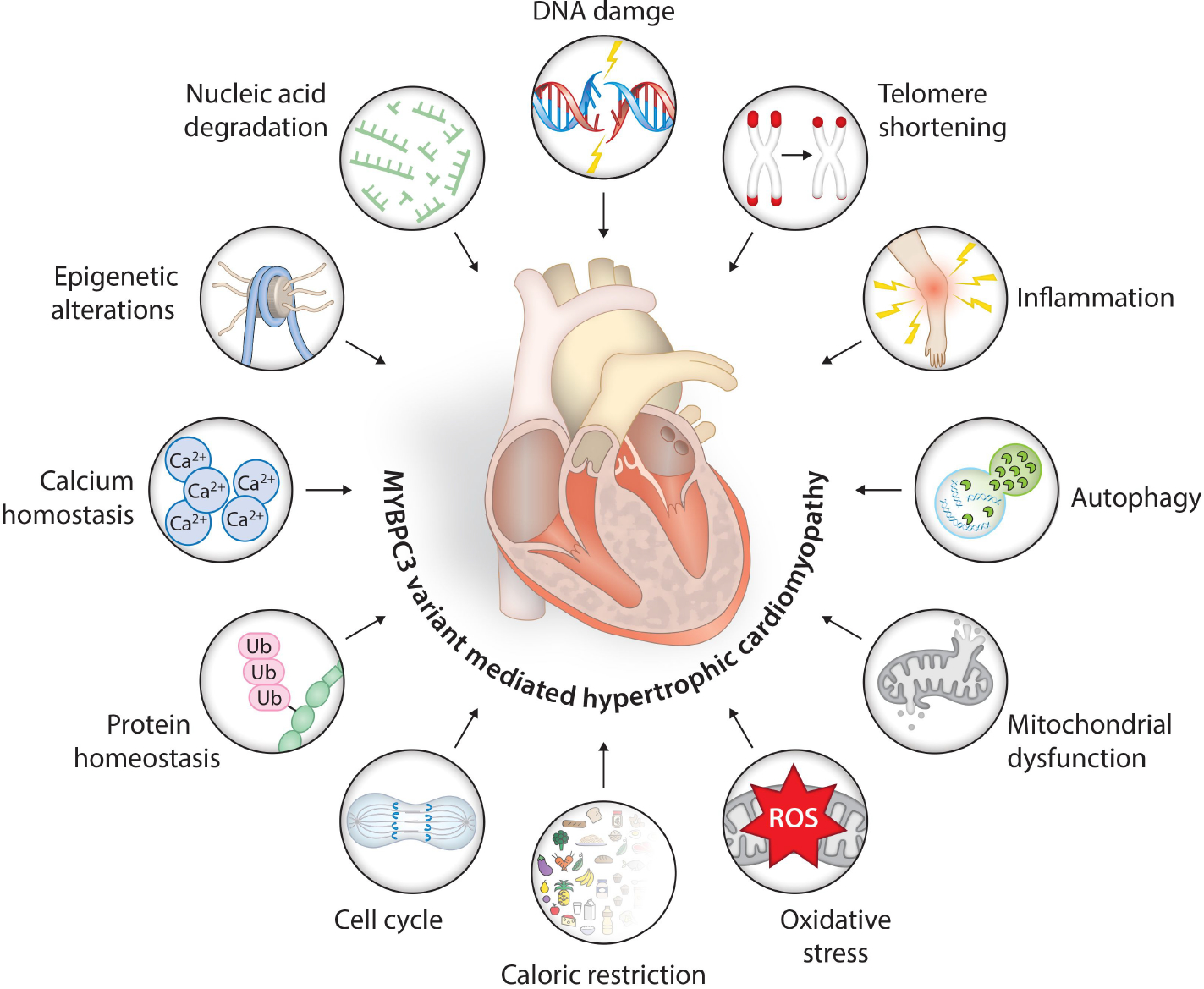
Schematic representation of *MYBPC3* gene variant-associated molecular events and age-associated hallmarks triggering hypertrophic signals in cardiac muscles. The nonsense-mediated mRNA degradation, ubiquitin proteosome-mediated protein degradation, alternate splicing, protein phosphorylation, and deregulated calcium sensing are the most common events in the HCM phenotype with *MYBPC3* mutations. The hallmarks of aging - namely, genomic instability, inflammation, autophagy, mitochondrial dysfunction, deregulated nutrient sensing, altered cellular senescence, protein homeostasis, and epigenetic alterations - activate aging-associated pathways in cardiac muscle cells. Both genetic mutation and aging-associated events generate pro-hypertrophic signals and induce hypertrophic phenotype affecting the cardiac muscle cell structure and function.

Aging, or cellular senescence, is a natural process whereby cells undergo alterations in morphology and function that, in turn, affect tissues, organs, and organisms. This process is characterized by reduced proliferation and regeneration capacity and increased cell death. Researchers have used long-lived mutants and mammalian animal models to understand aging-associated mechanisms. Senescence in cardiomyocytes involves increased expression of cell cycle inhibitors, proinflammatory cytokines, and senescence-associated β-galactosidase activity. Both mTORC1 and SIRT1 are primary molecules identified as regulators of lifespan^[56]^. Other hallmarks include oxidative stress, oncogene overexpression, caloric restriction, reactive oxygen species, DNA damage, protein homeostasis, shortened telomere structure, inflammation, and autophagy [Figure 3]. Current next-generation sequencing platforms help visualize the epigenomic profile of aging, revealing histone methylation as having a strong association with aging^[51,57–60]^.

#### Aging and cardiac function

**Figure 4.**
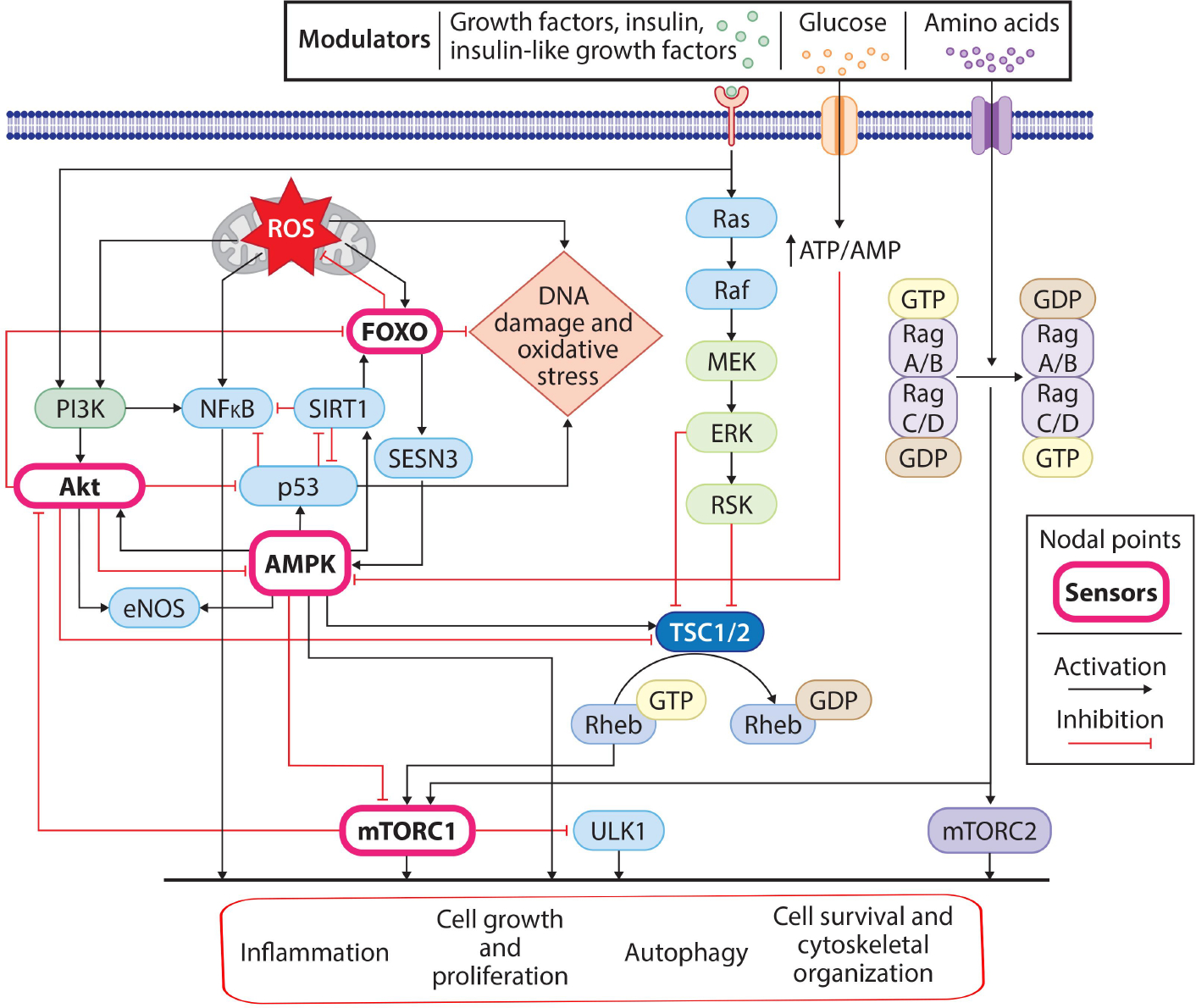
Key regulatory pathways involved in the aging process. Extracellular signals (nutrients, growth factors) and intracellular signals (genomic instability, mitochondrial dysfunction, and oxidative stress) initiate aging-associated signaling events. Cellular glucose uptake acts through the AMPK pathway, while growth factors, insulin, and insulin-like growth factors bind to their respective receptors and transmit signals through the Ras and PI3K-AKT pathways. Similarly, intracellular oxidative stress (ROS) and DNA damage-associated stress signals activate the PI3K, NFκB, SIRT1, and FOXO pathways. These pathways, either directly or through mTORC1, modulate cellular processes (elevated inflammation, reduced cell growth and proliferation rate, and inhibition of autophagy), leading to the aging phenotype.

Cardiac aging is a progressive decrease in ventricular function and increased ventricular and arterial stiffness accompanied by fibrosis stimulated by angiotensin II and proinflammatory cytokines. Key signaling pathways, including mitochondrial adaptor p66shc, AMP-activated kinase, sirtuins, insulin/insulin-like growth factor-1 signaling, and cAMP, are crucial in coordinating senescence, metabolic changes, and cardiovascular phenotypes [Figure 4]. SIRT1, a nicotinamide adenine dinucleotide (NAD)-dependent deacetylase, is present at elevated levels in a healthy heart. However, aging leads to the downregulation and reduced nucleocytoplasmic shuttling of SIRT1 in the myocardium, resulting in progressive cardiac dysfunction. Also playing a critical role in aging is insulin/insulin growth factor-1 signaling, which, when downregulated, increases lifespan^[61]^. Moreover, suppression of PI3K and its downstream effector mTOR improves protein turnover and prevents the accumulation of lipofuscin, an age pigment^[51,62–64]^.

A whole genome transcriptome and proteome microarray was performed to profile age-related gene expression differences in heart tissues. It revealed that inflammation-related genes were elevated, whereas genes involved in protein homeostasis, such as protein folding and the ubiquitin cycle, decreased with aging^[65]^. These intrinsic changes in cardiac systems increase the chance of pathogenesis of cardiovascular complications as age advances. From the perspective of heart dysfunction, apart from cardiac aging, pathogenic mechanisms in noncardiac tissues affected by aging also introduce additional risk factors, such as hypertension and diabetes. These factors contribute to the exponential increase in heart failure patients with advancing age^[66]^. Additionally, autophagy^[67]^, telomere shortening^[68,69]^, and DNA damage response^[70,71]^ act as molecular triggers of cardiac hypertrophy. Furthermore, a set of autophagy-related genes (ATG) regulates various types of autophagy, including macroautophagy, microautophagy, and chaperone-mediated autophagy, which are defined by their role in longevity or lifespan^[72]^. Among these genes, ATG5 has been linked to hypertrophy; cardiac-specific deletion of ATG5 resulted in hypertrophy, cardiac dilation, and contractile dysfunction, along with disorganized mitochondria, abnormal sarcomere structure, and accumulation of misfolded protein^[73]^.

In summary, the collective evidence listed above indicates that genetic defects in the sarcomere cause sarcomere dysfunction and disarray, which in turn lead to cardiac fibrosis, dysfunction, and LVH. Additionally, environmental factors, metabolic syndrome, and aging contribute to a diverse range of penetrance effects and the late onset of HCM. Pathways and molecular events associated with hypertrophy and aging processes exhibit shared characteristics. This is significant because the chronological or biological age of an individual could serve as a triggering factor for HCM, influencing the onset of disease phenotype, either early or late in life.

## *MYBPC3* GENOTYPE AND AGE-DEPENDENT INCOMPLETE HCM PENETRANCE

Mutations in the *MYBPC3* comprise approximately 50% to 60% of all mutations that have been identified in patients with HCM^[14,74–77]^. *MYBPC3* is the cardiac isoform (1273aa), which spans a 21kbp region with 35 exons. The other two skeletal isoforms, slow skeletal myosin binding protein-C (*MYBPC1*) and fast skeletal myosin binding protein-C (*MYBPC2*), share standard structural features, i.e., seven immunoglobulin domains and three fibronectin type III domains called C0–10 with myosin binding protein motif (M-domain) localized between C1 and C2^[78]^. The amino terminal ends C0, C1, and C2 and the M-domain can bind to F actin and S2 head regions of myosin heavy chain^[79,80]^. The carboxy-terminal C7-C10 binds to the C-Zone of myosin thick filament^[81]^. Interactions among titin, light meromyosin (LMM), and cMyBP-C occur in the C9-C10 region and C10 region, respectively [Figure 2]^[82]^. Additionally, cMyBP-C and myosin are arranged alternately, connecting thick and thin filaments. cMyBP-C is critical for regulating actomyosin function and cardiac contraction^[83–86]^. cMyBP-C acts as a brake by modulating cross-bridge kinetics, as determined by its phosphorylation status^[14,87]^. Approximately 75% of the mutations in the *MYBPC3* gene are truncating mutations, which include nonsense, frameshift (leading to insertions or deletions), and splicing (including branch point) mutations. The C′-truncated cMyBP-C protein lacks myosin LMM- and/or titin-binding sites in the C-terminus region, leading to its failure to bind with myosin and titin and incorporate into the sarcomere^[88,89]^. Nontruncated pathogenic variants, accounting for 25% of *MYBPC3* pathogenic variants, exhibit phenotypic effects like those of truncated variants. *MYBPC3* variants identified in some countries are population-specific and are classified as founder mutations, contributing to more HCM cases in the respective geographic location^[14]^. *MYBPC3* variants result in mild to moderate phenotypes with late disease onset^[48,90–93]^. The mechanisms underlying the late onset and development of HCM in *MYBPC3* carriers are still unclear.

### *MYBPC3* variants in elderly HCM patients

*MYBPC3* variants are consistently associated with clinical manifestations of HCM. The symptoms are more prominent in older individuals carrying *MYBPC3* mutants owing to incomplete penetrance. Among the sarcomere gene variants identified, *MYBPC3* mutations have been associated with delayed expression of HCM and favorable prognosis^[94]^. Often, HCM individuals with heterozygous *MYBPC3* loci have late disease onset with a non-threatening disease progression^[14]^. In addition, *MYBPC3* variants may display a DCM phenotype in a few cases, such as a 25bp deletion in intron 32 of *MYBPC3*^[90]^. A recent computational assessment of 73 non-truncating *MYBPC3* variants of uncertain significance predicted that 15 could affect RNA splicing, among which a few were experimentally confirmed using a minigene assay^[95]^. Considering the pathogenic potential of *MYBPC3* mutations and their significance in HCM, most population screening results estimate that 40%-60% represent genotype-negative individuals^[37,96]^. Recent studies reporting on cryptic gene variants of *MYBPC3* recommend including parallel gene sequencing for the entire *MYBPC3* region. Such parallel sequencing would include intronic gene variants and highlight the limitation of excluding splice region defects in current diagnostic HCM gene panels^[97,98]^.

HCM in elderly persons is genetically different from HCM in the unselected population^[31]^. An analysis of individuals 63 ± 11 years of age with late-onset HCM found that most harbored *MYBPC3* mutations, implicating these variants as the common factor underlying age-dependent HCM^[31,99]^. Yet another detailed familial study provided evidence for genotype-positive younger individuals with normal LV wall thickness; however, they had elevated wall thickness closer to midlife^[100]^. Another study concluded that *MYBPC3* mutation is more prevalent in younger people compared to the chronologically aged at the time of genetic testing. However, the genotype-positive elderly were significantly affected by the presentation of atrial fibrillation, systemic hypertension, and greater left ventricular dimensions compared with younger individuals. Furthermore, the crescent-shaped LV in genotype-positive elderly patients suggested the role of *MYBPC3* mutation as a true cause of late-onset HCM^[101]^. Many studies covering various geographic regions have defined the phenomenon of age-dependent incomplete penetrance in HCM gene carriers. These studies also attempted to establish the familial inheritance pattern by screening relatives. HCM and, in general, *MYBPC3* mutations are associated with later average age onset of symptoms, lower incidence of SCD, and benign clinical course. A few case studies emphasizing phenotype-negative diagnosis and age-dependent clinical outcomes, as highlighted in Table 2, suggest age-dependent penetrance among *MYBPC3* carriers.

To further define age as a factor contributing to HCM presentation in *MYBPC3* variants, heterozygous mouse models of familial hypertrophic cardiomyopathy (FHC) were generated with *Myh7* missense mutation and *Mybpc3* truncation mutation. These animals developed progressive hypertrophy in cardiac muscles, with *Myh7* heterozygous mice developing onsets as early as 30 weeks and exhibiting shorter life expectancies. In contrast, *Mybpc3* heterozygous mice developed prominent differences in cardiac biomarkers, but only after 125 weeks of age, a pattern similar to that observed in human carriers^[102]^. A novel isoform of mouse cardiac *Mybpc3* mutation has an additional 30 nucleotides that alter the splicing mechanism to generate mutant proteins. These mutant proteins created a disarray in the sarcomere structure, whereas their mRNA levels were predominantly expressed in the atria of aged mice, further implicating morphological and functional changes of cardiomyocytes during aging, i.e., age-dependent variation of cMyBP-C expression^[103]^. These studies provide strong evidence that gene variants in *MYBPC3* can worsen the clinical course of HCM with advanced age.

## MOLECULAR MECHANISMS UNDERLYING THE DEVELOPMENT OF HCM IN *MYBPC3* VARIANTS

The mechanisms behind the late onset and development of HCM in *MYBPC3* gene carriers remain unclear; nonetheless, the pathogenesis of familial HCM has been the focus of every mechanistic tier, from genotype to phenotype^[49]^. The two main mechanisms controlling the pathogenicity of *MYBPC3* gene variants are allelic imbalance and haploinsufficiency^[104,105]^. Allelic imbalance refers to a disproportion in wild-type and mutant allelic gene transcripts. In haploinsufficiency, when one copy of a gene is deleted or contains a loss-of-function mutation, the dosage of normal product generated by the single wild-type gene is not sufficient for complete function. It should be noted that translated wild-type protein is insufficient to meet the cellular requirement in cases of haploinsufficiency^[35]^. Epigenetic alterations can also play a significant role in developing hypertrophied cardiomyocytes^[53,106,107]^. In addition, recent studies provide evidence for the intercellular variation of myofilament cMyBP-C expression within the myocardium from HCM patients with heterozygous *MYBPC3* mutations^[108]^. Furthermore, alteration or mutations in the regulatory components, including transcription factors, miRNAs, splicing-associated proteins, proteins of nonsense-mediated decay (NMD), autophagy and the ubiquitin-proteasome system (UPS), as well as genotype-dependent differential expression of regulatory proteins, could also present a path forward in studying the molecular mechanisms underlying late onset of HCM in *MYBPC3* carriers [Table 3]. Therefore, continued intensive investigation of these pathways could finally reveal the molecular basis of genetic HCM.

### Transcription-dependent mechanisms

**Figure 5.**
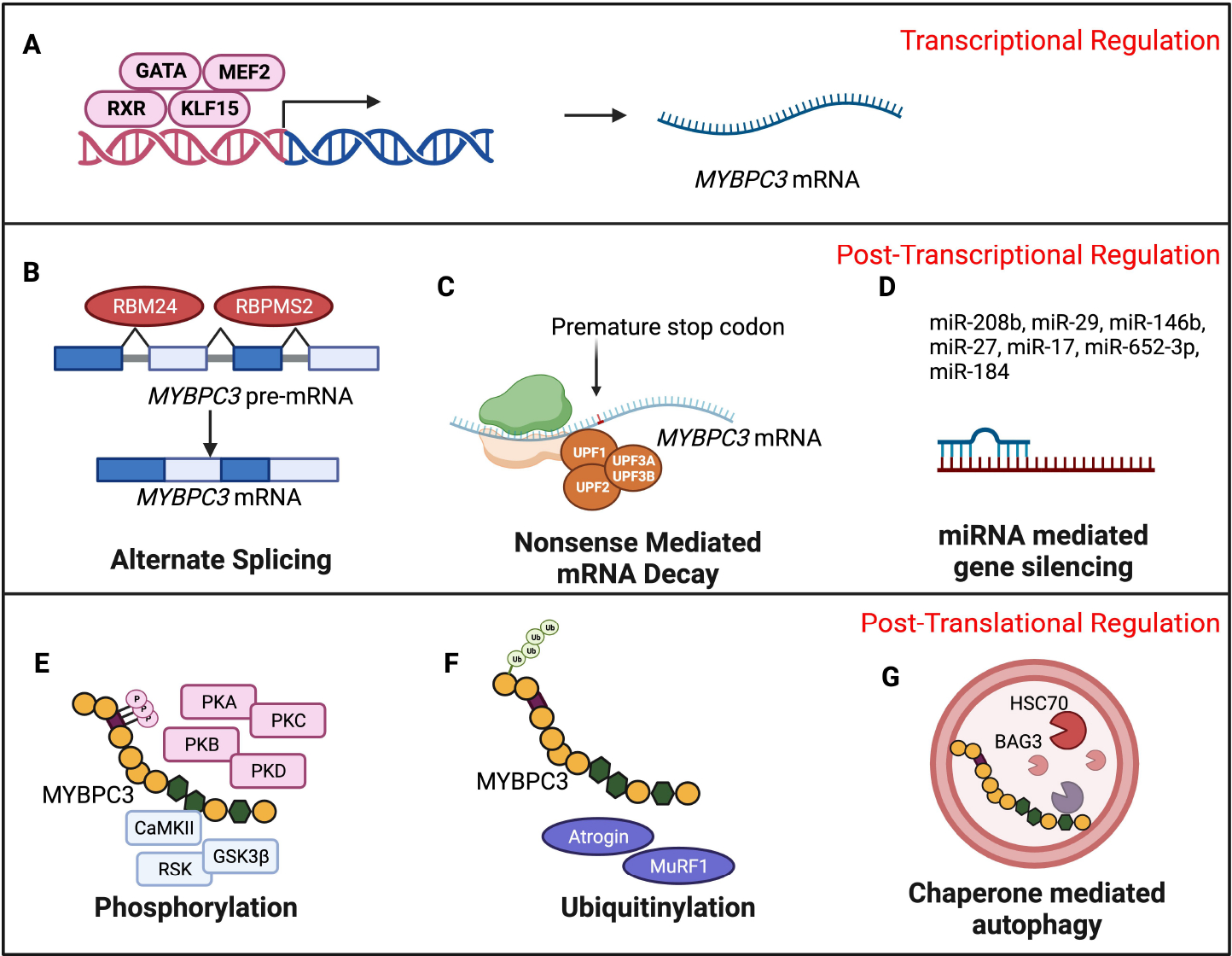
Potential HCM and aging-associated molecular events mediate the late onset of HCM among *MYBPC3* gene carriers. (A) Differential expression and enrichment of trans-acting factors in *MYBPC3* cis-elements regulate *MYBPC3* transcription upon aging. (B) Age-associated altered splicing mechanisms result in aberrant splice products, and (C) dysregulated mRNA degradation through nonsense-mediated pathways determines the abundance of *MYBPC3* mRNA available for translation. (D) Aging-induced differences in the expression of miRNAs might modulate *MYBPC3* translation through post-transcriptional gene silencing. (E) Age-dependent changes in phosphorylation, (F) ubiquitination proteasome system, (G) and chaperone-mediated autophagy determine the sufficiency of *MYBPC3* protein in preserving cardiac function (Figure Created with BioRender.com).

Transcription has been poorly studied for *MYBPC3*; however, computational prediction of the ~4 kb upstream promoter region of *MYBPC3* revealed potential binding sites for GATA and MEF2 transcription factors^[109]^. According to Akazawa and Komuro^[110]^, cardiac transcription factors have been directly associated with regulating cardiac sarcomeric genes and are involved in the development of cardiac hypertrophy, which is significant in cardiac muscle development and regulates cardiac specificity^[111,112]^. Differences in wild-type and mutant *MYBPC3* mRNA levels can be attributed to alterations in the interactions between trans-acting factors and cis-regulatory regions, either due to varying levels of transcription factors or epigenetic modifications at the *MYBPC3* promoter region [Figure 5A]. For instance, reduced *MYBPC3* mRNA levels were found in six genotype-positive HCM individuals with specific *MYBPC3* mutations compared to genotype-negative HCM individuals, suggesting a potential transcriptional regulation of *MYBPC3*^[110]^. Analysis in *MYBPC3* null mice revealed the involvement of Zinc finger and BTB domain containing 16 (ZBTB16) and other genes of HCM significance^[113]^. The role of GATA, MEF2, and ZBTB16 in *MYBPC3* expression remains unclear. However, multi-omics analysis in *MYBPC3* carriers identified several transcription factor binding motifs in hyper- and hypoacetylated regions, which need further confirmation for their role in *MYBPC3* transcription^[114]^. Additionally, increased methylation of *MYBPC3* in comparison to its skeletal isoform explains the genetic instability and increased occurrence of mutation in *MYBPC3*^[115]^. Although these epigenetic and genetic factors might explain the differential transcription level, their role in allelic imbalance needs to be verified. In line with the aim of this review, integrated transcriptomic and proteomic analyses might shed more light on age-related HCM in *MYBPC3* carriers.

### Post-transcriptional dependent regulation

Post-transcriptional regulation mainly involves RNA-binding proteins and non-coding RNAs, such as microRNAs, piwi RNAs, and long non-coding RNAs. These factors play roles in RNA processing, export, localization, turnover, and translation^[116,117]^. Among these, post-transcriptional control mediated by alternative splicing (AS), NMD, and post-transcriptional gene silencing (PTGS) in *MYBPC3* mutations is investigated next.

#### Alternative splicing

Alternative splicing is a vital process in RNA maturation, involving the removal of introns and the joining of exons. Splicing factors such as Serine/arginine-rich splicing factors (SRSFs), heterogeneous nuclear ribonucleoproteins (hnRNPs), nova proteins, transformer2 (TRA2), CUG-BP and ETR-3-like factor (CELF) proteins, and RNA-binding proteins (RBPs) play critical roles^[118–120]^. Splicing events lead to exon inclusion/skipping, intron retention, alternative splice site selection, and back splicing, generating circRNA molecules, stable and prevalent RNAs in eukaryotic cells that arise from back-splicing. Deregulation in alternative splicing events leading to the development of cardiac-related disease phenotypes has been recently reviewed^[121]^. In the context of cardiomyopathies associated with *MYBPC3*, cardiac dysfunction predominantly arises from splicing abnormalities stemming from mutations at donor or acceptor sites or the insertion and deletion of intronic sequences. Among *MYBPC3* mutations, splice donor variants constitute 1% and splice acceptor variants constitute 2%^[88]^. However, apart from conventional splice site mutations, screening of *MYBPC3* intronic variants identified four intronic variants in introns 9 and 13. These were predicted to cause cryptic splice sites in conventional splice site mutation-negative HCM patients. This signifies the importance of alternative splicing as a potential pathogenic mechanism in *MYBPC3* carriers [Figure 5B]^[98]^. Additionally, genetic defects in SRSF, hnRNPs, and RBPs have been implicated in severe and complex cardiomyopathies^[122]^. While defects in RBM20, a gene that encodes a protein that binds RNA and regulates splicing, have been identified as critical contributors to the development of both DCM and HCM, *MYBPC3* is not a substrate for it^[123,124]^. However, RBPMS2 has been identified as a splicing protein for *MYBPC3* in zebrafish^[125]^. Cardiomyocytes differentiated from human embryonic stem cells exhibited disrupted sarcomeric structures, the transcriptomic analysis of which revealed that *MYBPC3* was aberrantly spliced in RBM24 null phenotype^[126]^, explaining the role of these RBPs in processing *MYBPC3* mRNA.

#### Nonsense-mediated decay

Mature mRNAs transported to the cytoplasm with premature stop codons (PTCs) or transcripts lacking proper stop codons are degraded through nonsense-mediated decay (NMD) or non-stop decay mechanisms. The RNA surveillance system, which is initiated by the identification of premature termination codons, recruits several multiprotein complexes, including exon junction complexes composed of four core proteins and additional peripheral proteins that bind to 20–25 nucleotides upstream of the exon junction. Translation termination complex and release factors interact with up-frameshift factors (UPF), including UPF1, UPF2, UPF3A, and UPF3B. Phosphorylated UPF1 recruits the SMG-5/SMG-7 complex, triggering exo- and endonucleolytic degradation^[127]^. An estimated 70% of *MYBPC3* mutations are truncated with a premature termination codon^[128]^. Under experimental conditions, overexpression of C′-truncated cMyBP-C in cultured cardiomyocytes^[129,130]^ and mouse models^[131–135]^ showed the incorporation of mutant cMyBP-C into the sarcomere. However, there was no evidence of the presence of C′-truncated cMyBP-C in human biopsies from HCM patients^[136,137]^. Even though the NMD pathway acts as a primary pathway in degrading mutant mRNA in genetic diseases causing haploinsufficiency, mutant transcripts sometimes bypass the NMD pathway, resulting in the expression of truncated peptide causing poison polypeptide effects [Figure 5C]. In mice, *MYBPC3* mRNA with PTC mutations has cleared through the NMD pathway^[104,138]^. To date, studies evaluating the role of NMD genes in the degradation of mutant *MYBPC3* mRNA are limited. NMD inhibitors, such as emetine, a translational inhibitor, or cycloheximide (CHX), another NMD inhibitor, indirectly showed that mutant *MYBPC3* mRNA are cleared through NMD and escape this surveillance upon NMD inhibition^[104]^. Despite the difference in transcript levels, it has been recently discovered that protein levels were similar in cardiomyocytes cultured from isogenic lines of iPSCs derived from *MYBPC3* PTC variant p.R943x and its respective noncarriers^[139]^. Transcriptome analysis of these isogenic lines revealed that NMD pathway genes were activated, whereas cardiac signaling pathway-related genes were dysregulated, which was reversed upon inhibition of NMD^[139]^. However, a recent discovery identified UPF3B as a Z-disc-localizing protein involved in the degradation of truncated *MYBPC3* mRNA in left ventricular septum tissue from HCM patients carrying *MYBPC3* mutations (c.3288delG, c.2864_2865delCT, c.3697C>T, c.1458–6G>A, c1700_1701delAG, c.3490+1G> T, and c.927–2A>G)^[140]^. The accumulation of mutant mRNA evidence provides greater insight into the involvement of NMD surveillance in degrading the *MYBPC3* RNA isoforms generated by various types of mutations. This calls for further exploration of different NMD components in late-onset and disease heterogeneity of HCM in *MYBPC3* carriers.

#### Post-transcriptional gene silencing-mediated regulation

Among the many regulatory pathways of mRNA, PTGS has provided functional insights into the non-coding regions of the genome. PTGS mainly involves translation inhibition or mRNA decay through the recruitment of RNA-induced silencing complex (RISC) upon binding of miRNA^[141]^. No direct evidence can confirm the miRNA-mediated regulation of *MYBPC3* expression; however, independent studies observed differential miRNA expression in HCM individuals carrying *MYBPC3* mutations. The miRNA profiling of the endocardial interventricular septum regions of *MYBPC3* carriers with founder mutations c.927–2A>G (splice site mutation) and c.2373insG (truncation mutation) discovered that levels for 13 miRNAs were altered. Of these, 10 were upregulated (miR-181-a2*, miR-184, miR-497, miR-204, miR-222*, miR-96, miR-34b*, miR-383, miR-708 and miR-371–3p) and 3 were downregulated (miR-10b, miR-10a* and miR-10b*), suggesting crucial roles for PTGS in the initiation and progression of cardiac dysfunction^[142]^. In a similar study, plasma miRNA profiling of *MYBPC3* carriers with c.3369_3370insC and c.3624delC mutations reported that 385 miRNAs were upregulated, 279 miRNAs were downregulated, and 288 miRNAs were unchanged. About 33 miRNAs significantly differed in their expression levels (28 upregulated and 5 downregulated) between HCM and control subjects, forming the basis for establishing the significance of miR-208–3p in HCM^[143]^. These studies revealed notable alterations in miRNA profiles, thus highlighting the potential of miRNA-mediated pathways in understanding and potentially mitigating the complexities of genetically inherited diseases such as HCM [Table 1, Figure 5D].

### Post-translational modification-dependent regulation

After RNA processing and surveillance, mRNAs undergo translation, forming nascent polypeptide chains that enter the protein trafficking system and fold into tertiary protein structures. Stable proteins undergo enzyme-catalyzed PTMs on their side chains or backbones, enhancing their function. PTMs, including phosphorylation, ubiquitination, SUMOylation, O-GlcNAcylation, methylation, and acetylation, have implications in cardiac hypertrophy. These PTMs, triggered by various stimuli, activate or deactivate enzymes and signaling molecules, contributing to protein homeostasis and leading to changes in protein diversity, localization, structure, and interactions with other molecules. In cardiomyocytes, PTMs compensate for weak spatiotemporal transcriptional regulation and play a critical role in the cardiac hypertrophic process through intricate mechanisms^[144,145]^.

#### Protein modification-related mechanisms

Based on function and localization, protein molecules can undergo any one of a variety of covalent modifications, such as phosphorylation, acylation, alkylation, glycosylation, and oxidation, each one catalyzed by dedicated PTM enzymes. Various PTMs and their role in cardiac hypertrophy have been recently reviewed in detail^[145]^. Among these, protein phosphorylation by kinases constitutes a major PTM since most cellular processes rely on the phosphorylation status of its respective target protein. cMyBP-C, a thick-filament protein, exerts contractile function depending on its phosphorylation status^[84,146–150]^. The cMyBP-C protein has several phosphorylation sites, and the number of sites varies among species. For instance, human cMyBP-C has four phosphorylation sites, mice have three or four, canines possess five, and rats have three, all previously characterized for their roles in HCM^[85]^. An *in vivo* study identified N-terminal 17 residues confined to the N- terminal end of the protein (C0-C2) [Figure 2]^[151]^. The phosphorylation of cMyBP-C among these sites was essential to rescue cMyBP-C null mice, implying its crucial role in disease pathogenesis^[146]^. cMyBP-C phosphorylation sites are targets of protein kinase A (PKA; Serine 273, 282, 302, mouse sequence numbering)^[146,149]^, protein kinase C (PKC: Ser 273, 302)^[152–156]^, calmodulin-dependent kinase II (CaMKII; Ser 302)^[155]^, protein kinase D (PKD: Ser 302), and ribosomal S6 kinase (RSK; Ser 282). Additional phosphorylated sites Thr290 (mouse)^[157]^ and Ser 133 have been identified where Ser133 is predicted to be the target of Glycogen Synthase Kinase 3 Beta (GSK3β) [Figure 5E]^[158]^. Phosphorylated cMyBP-C is protected from proteolysis, but dephosphorylation has been associated with failing hearts. In spite of the presented evidence, the phosphorylation status of cMyBP-C remained unchanged in HCM patients with frameshift mutations in *MYBPC3* compared to their respective donor controls^[105,159]^. The contribution of cMyBP-C phosphorylation to late-onset HCM in gene carriers and the underlying mechanisms remain unclear. Apart from phosphorylation, acetylation of sites within a 40 kDA fragment of cMyBP-C was also determined to affect the contractility and function of the myofilament complex^[129,130]^. Similarly, S-glutathionylation of cMyBP-C in the heart tissue of a hypertensive mouse model resulted in the development of diastolic dysfunction^[160]^. The cMyBP-C protein is also subjected to calpain cleavage, citrullination, and s-nitrosylation^[161–163]^, all requiring further research to identify the significance of these mechanisms and their effects on HCM pathogenesis in *MYBPC3* carriers.

#### Protein degradation machinery

Protein levels undergo tight spatiotemporal regulation such that excess, unneeded protein or any misfolded and mutant proteins are targeted by the protein degradation system, either through the ubiquitin-proteasome system (UPS) or autophagy.

1. UPS: Proteins destined for degradation are modified by UPS-specific enzymes, i.e., E1, E2, and E3 ligases, in a reversible enzymatic reaction using ubiquitin as a substrate. The tagged proteins undergo proteolysis with the help of 26S proteasomes^[164–168]^. The second major HCM molecular mechanism observed among *MYBPC3* carriers was the dominant-negative phenotype owing to the generation of poison polypeptide^[133]^. Here, truncated cMyBP-C can be incorporated into the myofilaments, destabilizing the sarcomere. This leads to altered energetic mechanisms, activation of profibrotic pathways, constant stress, and premature death of the cardiomyocytes^[133]^. Further, in certain mutations, the lack of detectable mutant protein in heart tissue suggests that protein was not synthesized from mutant mRNA or was degraded rapidly, even if translated, eliminating the possibility of poison polypeptide-mediated pathway in the HCM phenotype^[169]^. Using inhibitors, such as epoxomicin (selective 20S proteasome inhibitor)^[104]^, bafilomycin (autophagosome lysosome inhibitor), and lactacystin (UPS inhibitor)^[170]^, it is evident that the cMyBP-C mutant/truncated protein undergoes substantial UPS-mediated degradation [Figure 5F]. Among various E3 ligases, muscle-specific E3 ligases are involved in activating proteostasis in cardiomyocytes^[171]^. Of note, atrogin-1/MAFbx (Z-disc) and the muscle ring finger proteins (MuRFs), including MuRF1-M band and MuRF-3-Z disc, are of particular interest by their localization in sarcomeres, and cMyBP-C acts as one of their substrates^[172]^. While atrogin-1 or MuRF levels remain unchanged in HCM hearts of *Mybpc3* knock-in mice, Asb2 E3 ligase is downregulated^[173]^.
2. Autophagy: This lysosomal-dependent protein degradation mechanism comprises macroautophagy, microautophagy, and chaperone-mediated autophagy^[174]^. Contrary to UPS-mediated cMyBP-C degradation, coimmunoprecipitation and mass spectrometry revealed that the Heat shock protein(HSP)-70 (HSP) family of chaperones interact with wild-type and mutant MYBPC3 protein, among which Heat shock cognate 71 kDa protein (Hsc70) was identified as an abundant interactor^[175]^. Notably, HSC70 is primarily involved in chaperone-mediated autophagy, whereby substrate proteins are targeted for lysosomal degradation by cathepsins^[176]^. The assembly of BAG-3 with HSC70 and HSPB8 to form a chaperone-selective autophagy (CASA) complex is crucial for cardiomyocyte contractility^[177]^. CASA complex helps stabilize the myofibrillar structure and inhibits myofibrillar degeneration under mechanical stress conditions^[178]^. BAG-3 expression was downregulated in DCM, and *MYBPC3* was one of eight myofilament proteins that interact with the CASA complex in the human heart^[177]^. Thus, the autophagy process involving BAG-3 and HSC-70 through sarcomeric protein quality control mechanisms and structural stability related mechanisms highlights the crucial role(s) of autophagy in the etiology of cardiomyopathy [Figure 5G]. Although these mechanisms have been investigated for several years, the role of protein degradation in the late onset of HCM in *MYBPC3* carriers remains unclear^[35]^.

## ASSOCIATION OF *MYBPC3* VARIANTS, AGING AND THE SEVERITY OF HCM

A systematic compilation of age-associated molecular changes in cardiovascular function has been recently published^[50]^. A close look at HCM pathogenetic mechanisms reveals that HCM processes are congruent with those of aging since they share some common hallmarks [Figure 3]. These include epigenetic changes, transcription factor activity, RNA processing and stability, transcriptional noise, and mitochondrial dysfunction. The major pathways in HCM with underlying causes of *MYBPC3* mutation include RNA metabolism, i.e., alternative splicing, NMD pathway, and protein metabolism (UPS). As discussed in the section below, age-associated aberrant splicing events, defective NMD, autophagy, and UPS systems might act as triggering events of HCM disease progression in *MYBPC3* carriers at older ages.

### Aging and alternative splicing

The effects of aging on transcription and translation processes have been a primary research focus for many years. Recently, however, alternative splicing, an intermediate step, has also gained attention for its significance in the aging process. The key finding is that aging increases isoform variations across species and tissues. Many components of the RNA processing machinery are themselves regulated by splicing. The differential expression of splicing trans-acting factors, aberrant RNA splicing, and aging genes provides mechanistic insights into splicing in age-dependent disease phenotypes^[179]^. Several studies have reported changes in splicing, e.g., reduction of unspliced transcripts and formation of more circular RNAs upon aging^[180–188]^. Additionally, the deregulation in splicing of genes involved in age-associated pathways, such as NFkB, mTORC1, and AMPK, plays a major role in the aging phenotype. These can be mediated through increase or decrease of specific isoform function, and imbalance in isoform ratio. Similarly, in trans-acting factor dysregulation, splicing factor levels also contribute to aberrant yields of splice variants. Interestingly, serine- and arginine-rich splicing factors SRSF3 and SRSF1 were reported to be significant in cellular senescence and longevity^[189–191]^. Alterations in splicing have been recognized in various tissues, and cardiac tissue-specific age-related changes in splicing factors have been reviewed in detail with a focus on cancer, neurodegenerative tissues, and progeria^[181]^.

More precisely, cardiac transcriptome and proteome analysis of young and early-aging mouse hearts determined widespread exon usage patterns compared to differential gene expression. The differential transcript levels of RNA-binding proteins and splicing factor-related genes were correlated with corresponding alterations in the levels of protein splice variants in the aging process^[192]^. Similarly, transcriptomic analysis of juvenile mouse versus adult cardiomyocytes, hepatocytes, and cerebral cortex found a higher expression of splicing factors, such as serine- and arginine-rich splicing factors (Srsf7, Srsf2), Y-box binding protein 1, heterogeneous nuclear ribonucleoproteins (Hnrnpa0, Hnrnpa1, Hnrnpdl) and splicing factor proline- and glutamine-rich (SFPQ) protein, in young mice^[193]^. Since alternative splicing is one of the molecular mechanisms of HCM in *MYBPC3* splice site variants^[128]^, age-related changes in alternative splicing events and splicing factors, as explained above, might contribute to the severity of HCM disease phenotypes. Cardiomyopathy-related RBM24 regulated p53 expression, which is known to induce aging-related heart failure^[194,195]^. Similarly, methylation-mediated RBPMS2 downregulation has been established^[196]^, and down-regulation of its mRNA in primary dermal fibroblasts has been associated with aging^[197,198]^. By elucidating the levels of RBM24^[126]^ and RBPMS2, we will be able to identify *de novo* pathophysiological mechanisms in age-associated HCM phenotypes.

### Aging and NMD pathway

The NMD pathway is key for maintaining RNA quality, but its role in the aging process is not yet well explored. Longevity mutants of animal models (C. elegans Insulin/IGF-1 receptor daf-2 mutant) help in understanding this phenomenon in the process of aging. In this model, silencing of NMD components suppressed longevity, showing that NMD protects the cells from undesirable proteins through its quality control. Both RNA-Seq analysis and mRNA half-life experiments suggested that enhanced NMD activity would prolong the longevity of the mRNA stability and translation. Overall, the reduction of the NMD pathway might hasten the aging process^[199]^. In addition, genetic analysis of RNAi feeding clones with lengthened lifespans identified smg-1 inactivation as a key factor in increasing the longevity of those clones. Interestingly, smg-1 is known to play a conserved role in the NMD pathway of worms and mammals^[200]^. The requirement of the NMD pathway has been implicated in the transition of cells from embryonic lineage towards differentiation. Hutch *et al.* identified an intricate connection between mRNA homeostasis and mTORC1 activity^[201]^. They show that NMD deficiency increased mTORC1 activity via the production of a premature termination codon (PTC) isoform of elongation initiation factor^[201]^. The mTORC1 is a key molecule in autophagy- and aging-related pathways, and its modulation through NMD clearly explains the involvement of the NMD pathway in lifespan. The involvement of mRNA decay in cardiac aging remains unknown. As previously mentioned in section 5.2.2, PTC mutation activates the NMD pathway^[130]^, whereas specific inhibition of the NMD pathway reverses the molecular phenotype and calcium handling abnormalities^[139]^. This might not be an effective treatment for the late onset of HCM, as the NMD pathway needs to be preserved to enhance the RNA surveillance mechanism during aging. However, further extension of research on animal models of aging will be crucial to determine the role of the NMD pathway and the RNA surveillance system in HCM disease with PTC mutations^[139]^. Thus, given a scenario wherein reduced NMD activity increases the senescence-associated phenotype, aging-associated elevated levels of mRNA and protein with *MYBPC3* PTC mutations might exacerbate HCM-related molecular changes. In the absence of scientific consensus, further exploration of NMD pathway dysregulation in the context of cardiovascular aging and its molecular consequences in genetic PTC mutations in *MYBPC3* carriers will shed more light on its significance in late-onset HCM.

### Aging and PTGS

Several studies using animal models and longevity experiments discovered that miRNAs are, indeed, a determinant of the aging process^[202–206]^. HCM miRNAs, as listed in Table 1, have been implicated in aging/elderly phenotypes^[207]^. A detailed review of key miRNAs involved in aging and cardiac aging is found elsewhere^[205,208–210]^. No evidence can be found of miRNA-mediated post-transcriptional gene silencing of *MYBPC3*; however, microarray of heart tissue samples and RNA sequencing of blood miRNAs have left us with few candidate miRNAs that are differentially expressed in HCM patients carrying *MYBPC3* variants. Among these identified miRNAs, miR-377, miR-200c, miR-208b, miR-103, miR-181–5p, miR-184, miR-96, miR-34, miR-383, miR-708, and miR-10 are all differentially expressed in the aged phenotype, such as the late passage cells, aged animal models and older individuals. Their involvement in regulating cellular senescence and longevity mechanisms has been implicated in different tissues [Table 1]. Further validation of these miRNAs in heart tissues of aged cells/animals or human disease models will help uncover novel roles for miRNA-mediated HCM and its age-dependent HCM penetrance in *MYBPC3* carriers.

### Aging and post-translational regulation of cMyBP-C

Chronic -adrenergic receptor (-AR) stimulation can result in the development of left ventricular hypertrophy and HF^[211]^. Novel therapeutic strategies, such as direct activation^[212,213]^ and/or inactivation^[214]^ of myosin, are needed to ameliorate the side effects associated with current pharmacological therapies. -AR stimulation activates protein kinase A, which phosphorylates sarcomeric myofilament proteins, including cMyBP-C. cMyBP-C undergoes other post-translational modifications (PTM), including ubiquitination, SUMOylation, O-GlcNAcylation, methylation, carbonylation, and acetylation^[215,216]^. However, a systematic study is required to link the PTM of cMyBP-C to the development of HCM^[217–219]^.

#### Post-translational modification and aging

cMyBP-C phosphorylation is a significant phenomenon in age-dependent incomplete penetrance of HCM^[147]^. Phosphorylation and PTMs affect cardiac function^[85]^ and the status of PTM processing, along with the activity of various enzymes involved in these modifications upon aging, might be predictors of the pathophysiological mechanisms behind age-dependent incomplete penetrance in *MYBPC3* carriers. For example, mice lacking the regulatory subunit of protein kinase A (PKA) complex have extended lifespans and are resistant to cardiac dysfunction, which explains its significance in aging and cardiac function. Previous literature identifies PKA as a target for aging and the aging heart and proposes it as a therapeutic target to rescue aging phenotypes^[220,221]^. Similarly, the deregulation of c-Jun NH2-Terminal kinase (JNK), CaMKII^[222]^, and Protein Kinase B (PKB)^[223,224]^ in animal models of aging has been ascribed to the reduced immune and neurological functions in the elderly, and these kinases are considered biological markers of aging.

To understand the pathophysiological mechanism in age-dependent pleiotrophy, cMyBP-C phosphorylation mechanisms have been investigated extensively. A total phosphorylation quantitation of myofilament proteins in the hearts of naturally aging mice determined that the phosphorylation status of cMyBP-C increased with age in both sexes^[225]^. Additionally, this study determined that cMyBP-C phosphorylated at Ser^295^ and Ser^315^ positions was increasingly expressed in neonatal hearts compared to adult hearts. These findings suggest a protective role in the neonatal heart against hypoxia and acidosis^[226]^ and establish a possible correlation between aging, heart disease, and the phosphorylation status of cMyBP-C. To further understand, wild-type, phospho-mimetic (mutated at Ser^273^, Ser^282^, Ser^302^ sites to aspartic acids), and phospho-ablated cMyBP-C (mutated at Ser^273^, Ser^282^, Ser^302^ sites to alanine), transgenic mice were maintained for 18–20 months^[227]^, mimicking the aging of human subjects (60–70 years). Among these, phospho-mimetic cMyBP-C mice had better survival and better preservation of systolic and diastolic functions, as well as constant wall thickness, compared to wild-type and phospho-ablated cMyBP-C mice. This evidence suggests that phosphorylation of these residues is beneficial in protecting age-related cardiac dysfunction^[227]^. In the context of *MYBPC3* variants, genotype-positive HCM patients with R1073W, E542Q, D770N, E461X, L527fs/3, Q791 fs/40, and V1063 fs/63 mutations exhibited diminished phosphorylation at their myosin regulatory light chain compared to noncarriers^[105,228]^. While the presence of *MYBPC3* mutations appears to alter phosphorylation levels, more detailed studies are needed to validate their modulations, along with the pathological consequences in late-onset HCM.

#### Protein quality control and aging

In age-associated severity of HCM, cardiomyocytes undergo a variety of molecular changes, among which UPS could play a major role. To understand the role of UPS in late-onset HCM, age-associated UPS dysfunction needs to be addressed. Dysfunction in protein synthesis and degradation alters the steady state of the macromolecular degradation system and imparts enhanced ER stress and inflammatory signals^[229]^. The involvement of UPS is well recognized in senescence and aging events, viz., NF kB signaling, AMPK-activated protein kinase activity, cyclin-dependent kinase and inhibitors, and FOXO transcription-mediated protein processing^[230]^. Aging-dependent biological functions, such as immune and inflammatory response, cellular metabolism, autophagy, and cellular proliferation, also require extensive protein processing, in part from UPS.

Among the many pathways defined for cardiac diseases, macromolecular dynamics have been considered closely related to cardiac aging. Among the protein and nucleic acids dynamics, reduced protein degradation pathways are the major pathway discussed in many age-related phenotypes, including cardiac aging and HCM^[170,230–233]^. Using the longest-living noncolonial animal, Arctica islandica, it was concluded that maintaining protein homeostasis is essential to the preservation of cardiac function and that low-grade chronic inflammation in the cardiovascular system is a hallmark of the aging process^[233,234]^. Similar results were observed in Fisher 344 rat hearts, where proteasome activity was shown to decrease with age, thus resulting in the loss of proteasome function. Consequently, dysregulation of protein homeostasis results in ubiquitinated protein accumulation in the aged cardiomyocytes and heart^[62,66]^. Abnormal inclusion bodies are characteristic of neurodegenerative disease, where impaired protein homeostasis plays a major role. Similarly, cardiac amyloids were also detected in patients with amyloidosis^[235]^. The development of HF was also associated with inappropriate protein metabolism. These events led to the inability of cardiomyocytes to elicit an appropriate response to age-related stress and associated cardiovascular complications.

As cMyBP-C variants are subjected to UPS-mediated protein degradation, it is reasonable to presume that an age-dependent decline in UPS might result in enhanced mutant protein production at later ages. Indeed, the same phenomenon has been defined experimentally where impairment in the UPS system was observed in *MYBPC3* mutant genotypes^[170]^, as well as UPS saturation or impairment in older *MYBPC3* knock-in mice^[236]^. Although HSC70, a chaperone-mediated autophagy-related protein, was recognized as the abundant protein interactor of *MYBPC3*, mutations in *MYBPC3* did not affect HSC70 localization, nor did it induce a protein folding stress response or ubiquitin proteasome dysfunction^[175]^. However, dysfunction in chaperone-mediated autophagy was well documented in aging^[176]^; notably, HSC70 and BAG3 have been defined as having a potential role in aging^[237–240]^. As discussed above, alterations in the proteasome and chaperone-mediated autophagy are involved in aging, senescence, and regulation of lifespan, and their decline in function has been attributed to advancing age. These findings indicate that the enhanced existence of mutant protein at older ages results in poison polypeptide and dominant negative mechanisms, increasing the severity of HCM phenotypes in older populations.

## CALCIUM SENSITIVITY IN HCM AND AGING

Cardiomyocyte functions depend on the force generated by myofilaments. Myocyte calcium concentrations are essential for actin-myosin interactions in the myofilaments. Intracellular calcium reserves in the sarcoplasmic reticulum (SR) and sarcomeric units are released upon electrical stimuli and are sequestered back to restore the calcium in sarcoplasmic reticulum (SR) through Ca^2+−^ATPase^[241]^. The excitation-contraction coupling process involves cell membrane excitation and subsequent contraction by an action potential. The action potential resulting in cell membrane depolarization is followed by a rise in intracellular calcium levels. The calcium ions released from the endoplasmic reticulum attach themselves to the calcium-binding component of troponin, known as troponin-C (TnC), whose interaction counteracts the inhibitory effects of troponin, leading to the initiation of muscle contraction. Tropomyosin can now move along the actin surface and thus activate contraction^[109]^. However, any change in calcium concentrations results in calcium mishandling, leading to perturbed force generation and pathological conditions of cardiac diseases^[242–244]^. The role of calcium in cardiomyocyte function, calcium signaling pathways, and many associated genes and proteins, along with their significance in cardiac dysfunction, has been reviewed in detail elsewhere^[245]^.

### Calcium sensitivity in MYBPC3 carriers

Abnormal calcium (Ca^2+^) homeostasis is considered one of the molecular mechanisms underlying HCM. Current pharmacological treatments for HCM often include calcium channel blockers, which reduce the availability of Ca^2+^ and inhibit contractions. In HCM, an increase in calcium sensitivity is recognized as a significant hallmark event. A detailed analysis of either mutation in *MYBPC3* (c.1358dupC, c.1960C>T, and c.2308G>A) alone or along with mutations in other genes, such as Filamin C (FLNC) (c.2234A>G), Lysosomal Associated Membrane Protein-2 (LAMP2; c.1988G>A) or MYH7 (c.1293C>T), revealed that compound mutations exhibited reduced contractile force (F_max_) with HF compared to single mutations in *MYBPC3*. However, calcium sensitivity was most pronounced in compound mutations with *MYBPC3* mutations^[246]^. Further analysis of cardiac tissue samples from individuals carrying 15 different *MYBPC3* mutations associated with HCM showed elevated protein levels of CaMKIIδ, along with increased phosphorylation of pT17-phospholamban, a target of CaMKIIδ. However, *MYBPC3* mutant-mediated HCM was not rescued by crossing the mice with phospholamban knockout mice^[247]^. The levels of histone deacetylase (HDAC4) were elevated in the HCM, suggesting a possible epigenetic process activated by CamKII^[248]^. These findings provide insights into the effects of cMyBP-C mutations and their association with heightened calcium sensitivity in myocytes of patients with gene variants. Similar results have been observed in studies involving the removal or deficiency of cMyBP-C in myocardial samples, further supporting the link between cMyBP-C mutants and increased calcium sensitivity, which has been extensively reviewed elsewhere^[249]^.

### Calcium homeostasis in aging

Calcium homeostasis is critical for healthy heart function, and its disturbance causes various cardiac complications, including the HCM phenotype^[105,250–252]^. Furthermore, higher calcium sensitivity, as observed in *MYBPC3* carriers^[105,250]^, is requisite to define its role in late-onset HCM in these individuals^[76]^. Based on earlier publications, a decrease in calcium transient amplitude has been recognized as a well-known phenomenon associated with age^[225]^. As age advances, evidence suggests an increase in diastolic and systolic dysfunction. The role of age in cardiomyocytes with respect to calcium homeostasis and contractile functions has been reviewed in detail by Feridooni *et al.*^[253]^. Increased sensitivity to calcium and incidence of diastolic dysfunction are early indicators of *MYBPC3* mutation-mediated HCM^[250]^, which is also a major phenomenon associated with advanced age. Alterations in calcium sensitivity have been established in *MYBPC3* mutant carriers, but evidence also suggests that calcium handling is not likely to be deregulated in *MYBPC3* mutations where alterations in contractile kinetics are independent of calcium flux^[169]^. Consistently, the lack of correlation between calcium and mutant background has been proven using 3D cardiac tissues generated from hiPSC of *MYBPC3* carriers^[254]^ and phosphorylation site mutant transgenic mice^[255]^. With further research to ascertain the significance of calcium signaling in *MYBPC3* carriers, age-dependent calcium dynamics might be confirmed as a potential cause of HCM penetrance in the elderly.

## CURRENT TREATMENT OPTIONS

Current research is focused on the correction of sarcomere deficit and rescue from HCM phenotypes. Strategies include gene replacement or gene silencing by anti-sense oligonucleotides or CRISPR-based gene modification, which is in the preclinical stage^[256–258]^. CRISPR Cas-based gene modification is in the proof-of-concept stage for *MYBPC3* mutations^[259]^. At the cellular energetics level, optimizing cardiac metabolism and mitochondrial function can be targeted as a potential therapeutic intervention. This strategy includes small molecules targeting different sarcomere functions, such as calcium sensitivity, cross-bridge kinetics, tension cost of myofilament, ATP utilization, and super-relaxed state. MYK-461 (mavacamten, myosin inhibitor) has completed its preclinical and clinical trials and is available in the clinic for HCM treatment^[260–262]^. Since PTC mutations are the primary activators of the NMD pathway, several other cellular dysfunctions and associated disorders are also affected by cellular NMD, such as senescence and aging. Detailed profiling of FDA-approved drugs for their impact on NMD efficiency identified significant NMD targets and NMD modulators. For example, the anticancer drug homoharringtonine (HHT or omacetaxine mepesuccinate) increased NMD substrates in multiple cell types with concomitant increase in NMD processing^[263]^.

Antagomirs, also known as anti-miRs, for miR-132 as a treatment for HF^[264,265]^ and anti-miR-92 for the treatment of cardiovascular disease and wound healing^[266]^ have been developed, and both have entered preclinical and clinical trials. Considering this, the validation of miRNAs that regulate both wild-type and variant forms of *MYBPC3* holds promise as a potential therapeutic approach for HCM in individuals carrying *MYBPC3* mutations. For example, Tenaya Therapeutics is conducting clinical trials involving the overexpression of cMyBP-C in patients with HCM (TN-201, Clinical Trial ID: NCT05836259). Apart from these, focusing on age-related pathways could also reduce the effects of age-dependent increase in HCM severity. Interventions, such as caloric restriction and intermittent fasting, expression of cardiomyocyte-specific dnPI3K and transgenic overexpression of Parkin, low to moderate SIRT1 in transgenic mouse hearts^[267]^, protein kinase B β (Akt2) knockout, spermidine, Rapamycin, Resveratrol, and SRT1720 treatment, have resulted in modulating SIRT1, mTOR, AMPK and FOXO pathways and delayed cardiac aging, thus preserving cardiac function in aged animal models^[268]^. A few other common drug options include Metformin, ACE inhibitors, Aspirin, Statins, β-blockers, AT1 blockers, Omecamtiv mecarbil, Berberine, polyunsaturated fatty acids (PUFAs), NFE2 like bZIP transcription factor 2 (Nrf2) activators and Mito-targeted antioxidants^[269]^. With a growing number of drugs and treatment options, demethylation agent 5-azacytidine, displaying antifibrosis and anti-hypertrophic properties^[270]^, will be a promising strategy to prevent hypermethylation of CpG islands of *MYBPC3*. Thus, along with the available advanced diagnostic and therapeutic interventions, nondrug-based (diet and physical exercise) or drug-based modulations of gene regulation and signaling pathways related to hypertrophic phenotype and aging could afford elderly *MYBPC3* carriers a better chance of early diagnosis and prevention of HCM severity.

## CONCLUSIONS

The age-dependent incomplete penetrance of *MYBPC3* mutations is characterized by heterogeneous clinical presentation mainly affecting elderly carriers, whereas younger individuals experience SCD more often. *MYBPC3* mutant mRNA and protein are processed by NMD and UPS systems, causing haploinsufficiency or deficiency and a deficit in sarcomere function. This disarrayed sarcomere structure and function trigger a stress-sensing mechanism that causes cellular perturbations, such as changes in epigenetic factors and altered signaling pathways, leading to the initiation of the hallmark events of cardiac hypertrophy. To preserve functional myofibrillar components and cardiac function, it is essential to identify modulators of *MYBPC3* mutant gene and protein expression like epigenetic factors, transcription factors, and proteins associated with AS, NMD, and UPS pathways. Dysregulation of any of these checkpoints would have a significant impact on the onset of HCM disease phenotype. Age-associated alterations of *MYBPC3* modulators also play a role in triggering increased severity of HCM in the elderly.

Some potential treatment options have emerged at various levels of preclinical and clinical trials. Needless to say, strategies aimed at ameliorating the severity of HCM in the elderly require additional focus on identifying potential biomarkers, therapeutic targets, and novel drugs. High-throughput RNA sequencing, small RNA sequencing, and ChIP sequencing in elderly human HCM tissue, as well as animal models of human aging, will be valuable tools in the selection of key target molecules, genes, and pathways for therapeutic intervention^[271]^.

## Supplementary Material

Supplementary Material

## Figures and Tables

**Table 1.** List of miRNAs deregulated in *MYBPC3* variant samples and their association with cardiac disease phenotype and aging

S. No	miRNA	MicroRNA expressions in HCM	Age/senescence/longevity-related changes in microRNA expression

1	miR-377-3p	Peripheral blood miRNAs-upregulated in HCM patients c.3369_3370insC and c.3624delC *MYBPC3* mutation^[143]^	Upregulated in late passaged human skin^[272]^; miRNA inhibition reduces senescence and increases cell viability^[273]^
2	miR-103a-3p		Lower in peripheral mononuclear cells of older individuals^[203]^
3	miR-146b-3p		Upregulated during senescence^[274]^; lower expression levels in bronchial tissues of elderly^[275]^ and aged macrophages^[276]^ downregulated in perivascular adipose tissue of aged mice with cold stimulus^[277]^; upregulated in aging heart^[278]^
4	miR-208b		Upregulated inyoung mice compared to older ones under post-traumatic osteoarthritis^[279]^
5	miR-200c		Upregulated in aging liver, aged skin[280,281]; upregulation in endothelial cell death and senescence

6	miR-181-5p	Cardiac miRNAs-upregulated in cardiac tissues of HCM patients with c.927–2A>G, c.2373insG mutations^[142]^	NK cell aging[282,283], age-related loss of muscle cells^[284]^, and skeletal cell aging^[285]^; downregulated in peripheral blood and associated with all-cause mortality and age-related traits[286]
7	miR-184		Downregulated in calorie-restricted mouse brain^[287]^, older adults with major depressive disorder^[288]^, downregulated in aging heart^[278]^
8	miR-222-5p		Altered copy number in aging^[289]^
9	miR-96		Increased with age in peripheral blood mononuclear cells^[290]^
10	miR-204		Upregulated in aged hippocampus^[291]^
11	miR-10a	Cardiac miRNAs-upregulated in cardiac tissues of HCM patients with c.927-2A>G, c.2373insG mutations^[142]^	miR-10b, decreased in PD and increased in HD; both are age-associated diseases[292,293]; downregulated in liver that senesces with aging^[294]^
12	miR-10b		MiR-10a*/miR-10–3p enriched in exosomes derived from frail, older individuals^[295]^

13	miR-652-3p	Myocardial miRNA upregulated in HCM^[296]^	Upregulated in aging heart^[278]^
14	miR-17-5p	HCM cardiac tissue upregulated miRNA^[297]^	Upregulated in aging heart^[278]^
15	miR-29a	Plasma miRNAs upregulated in HCM^[298,299]^	Upregulated in aging heart^[300,301]^
16	miR-1–3p	Downregulated in Left ventricular heart tissues^[302]^ and HCM	Elevated levels in colon tissues of older individuals^[303]^
17	miR-27a		Upregulated in aging heart^[278]^
18	miR-155	Plasma miRNA downregulated^[298]^; upregulated^[302]^ in HCM/DCM	Lower in older individuals^[203]^

**Table 2.** *MYBPC3* genetic mutations familial case studies presenting age-dependent penetrance

*MYBPC3* mutation	No. of gene carriers	Age of probands during screening and their clinical presentation

c.2833_2834del, p.Arg945fs	4 of 176 HCM probands, 1 of 54 DCM probands, 1 relative of these 5 probands	61 ± 10 years (48–79 years); LV systolic dysfunction and suffer from cardiovascular events midlife and beyond^[304]^
c.2373dup, p.Trp792fs	27 of 49 probands; only 10 were symptomatic, 5 with borderline, and 12 asymptomatic	29–69 years; symptomatic HCM underscored by more advanced age of 54.7 ± 4.5 years; borderline HCM or unaffected carriers were 40 ± 14.0; non-gene carriers were 43 ± 12.9; asymptomatic at 37.8 ± 13.6^[137]^
c.2459G>A, p.Arg820Gln	8 probands (7 HCM, 1 DCM) of 250 HCM probands and 90 DCM probands. Among 24 relatives of these 8 probands, 17 were genotype-positive	16–77 years; burnout phase of HCM (patients with over dysfunction defined by an LVEF < 50%^[11]^) was described in those > 70 years of age The disease penetrance was 70% in subjects > 50 years of age by echocardiography and 100% by ECG, and in those aged < 50 years, it was 40% and 50%, respectively^[305]^
c.1777del, p.Ser593fs, (V592fs/8c)	15 of 94 probands, 24 relatives of these 15 probands were genotype-positive	48 ± 14 (16–83); 100% disease penetrance; in ≥ 50 years; 65% disease penetrance among individuals < 50 years^[92]^
g.47332282_47332306del	49 (13.8%) carried the 25-bp deletion (46 heterozygotes and three homozygotes) of 354 cases	48 ± 8 (Group 1 case) and 49 ± 12 (Group 2 case). Symptomatic carriers 55.7 ± 15.3 years; asymptomatic carriers 40 ± 14.1 years among 120 family members of 28 affected families with cardiomyopathy; 90% of the oldest members are symptomatic, whereas young and middle-aged are asymptomatic^[90]^
c.1000G>A, p.Glu334Lys	9 of 1017 unrelated probands were gene carriers	15–76 years; Delayed clinical presentation, reduced penetrance of HCM in women than men^[306]^
c.2308 + 1 G>A, pro108Alafs*9	13 probands and their relatives of a total of 107 individuals were screened; among the 54 gene carriers, 39 had HCM	Male-50.5 ± 15.9, female-65.5 ± 17.4 years; lower penetrance rate and later onset in women than men^[307]^

**Table 3.** Aging effect on potential molecular regulators of *MYBPC3* and HCM

	Age/senescence/longevity-related changes

**Transcriptional regulators**	
Transcription factors	
GATA	GATA4 accumulates in senescent cells^[308]^; GATA6 expression was decreased in younger mice or later passaging of cultured mesenchymal stem cells^[309]^
MEF2	Age-dependent downregulation of MEF2 in microglial cells; rescues retinal explant culture from age-dependent photoreceptor degeneration^[310]^
KLF15	Downregulated in aged subcutaneous white adipose tissue^[311,312]^
ETS2 (ETV4)	Elevated levels of ETS2 promoted apoptosis-inducing factor-mediated programmed necrosis^[313]^
RXRA	Deficiency results in cellular senescence. RXR pathway genes decreased expression in aged macrophages in monocytes of multiple sclerosis patients. RXR receptors and other nuclear receptor factors regulate cellular senescence^[314–316]^
**Post-transcriptional regulators**	
Alternative splicing	
RBM24	Cardiomyopathy-related RBM24 regulated p53 expression, which is known to induce aging-related heart failure^[194,195,317]^
RBPMS2	Age-associated decreased mRNA expression in primary dermal fibroblast^[197,198]^
SRSF3 and SRSF1	Cellular senescence and longevity^[189–191]^
Srfs7, Srsf2, Y box binding protein, 1 Hnrnpa0, Hnrnpa1, Hnrnpd1, Sfpq	Elevated expression in young mice^[193]^
**NMD Pathway**	
UPF1	SMG-2/UPF1 promotes longevity in worms (C. elegans)^[199,318]^
UPF3 (UPF3A and UPF3B)	Deletion of UPF3 decreased the mean chronological lifespan in yeast^[319]^. UPF3B is associated with age-related disorders^[320]^
**Post-translational regulators**	
Protein modification	
PKA	Mice lacking the PKA gene have extended lifespans and resistance to cardiac dysfunction^[220,221]^
PKB, JNK, CaMKII	Deregulated in animal models of aging^[222–224]^
Calpain	Calpains are activated in aging^[321]^. Calpain inhibitors improve/reverse age- associated damage in heart function ^[322,323]^
Protein degradation	
Atrogin-1 (MAFbx) and MuRF1 (Trim63)	Differential expression in age-associated sarcopenia, muscle wasting, and diet restriction in rats and human muscle tissues^[324–330]^; elevated levels of atrogin-1 reduce age-associated cardiac fibrosis^[331]^, and its deficiency promotes premature death, cardiomyopathy, and heart failure^[332]^
ATG-5	Cardiomyocyte-specific constitutive knockout of Atg5 enhanced susceptibility to pressure overload. Cardiac- specific Atg5 knockdown mice exhibited age-related cardiomyopathy. Podocyte-specific knockout caused spontaneous age-dependent late-onset glomerulosclerosis. ATG5 is downregulated in normal human brain aging. ATG5 transgenic mice are lean and have extended lifespans. Embryonic fibroblasts cultured from ATG5 transgenic mice are tolerant to oxidative stress and damage^[73,333–336]^
HSC70	Decreased LAMP-2A and Hsc70 protein levels in the muscle of aged mice remain unchanged in heart. Decrease in levels in olfactory bulbs^[238,240]^
BAG-3	Switching from BAG-1 to BAG-3 in aging BAG-3/BAG-1 ratio elevated in neurons during aging of rodent brain ^[237,239]^

## References

[1] Semsarian C, Ingles J, Maron MS, Maron BJ. New perspectives on the prevalence of hypertrophic cardiomyopathy. J Am Coll Cardiol 2015;65:1249–54. 25814232 10.1016/j.jacc.2015.01.019

[2] Kramer CM, Appelbaum E, Desai MY, Hypertrophic cardiomyopathy registry: the rationale and design of an international, observational study of hypertrophic cardiomyopathy. Am Heart J 2015;170:223–30. 26299218 10.1016/j.ahj.2015.05.013 PMC4548277

[3] Wolf CM. Hypertrophic cardiomyopathy: genetics and clinical perspectives. Cardiovasc Diagn Ther 2019;9:S388–415. 31737545 10.21037/cdt.2019.02.01 PMC6837941

[4] American College of Cardiology Foundation/American Heart Association Task Force on P, American Association for Thoracic S, American Society of E, 2011 ACCF/AHA guideline for the diagnosis and treatment of hypertrophic cardiomyopathy: a report of the American College of Cardiology Foundation/American Heart Association Task Force on Practice Guidelines. J Thorac Cardiovasc Surg 2011;142:e153–203. 22093723 10.1016/j.jtcvs.2011.10.020

[5] Marian AJ, Braunwald E. Hypertrophic cardiomyopathy: genetics, pathogenesis, clinical manifestations, diagnosis, and therapy. Circ Res 2017;121:749–70. 28912181 10.1161/CIRCRESAHA.117.311059 PMC5654557

[6] Ramchand J, Fava AM, Chetrit M, Desai MY. Advanced imaging for risk stratification of sudden death in hypertrophic cardiomyopathy. Heart 2020;106:793–801. 31949025 10.1136/heartjnl-2019-315176

[7] Lai EJ, Rakowski H. Physiologic or pathologic hypertrophy: how can we know? Expert Rev Cardiovasc Ther 2014;12:919–22. 24972675 10.1586/14779072.2014.934226

[8] Li Q, Gruner C, Chan RH, Genotype-positive status in patients with hypertrophic cardiomyopathy is associated with higher rates of heart failure events. Circ Cardiovasc Genet 2014;7:416–22. 24909666 10.1161/CIRCGENETICS.113.000331

[9] Kaplinsky E. Significance of left ventricular hypertrophy in cardiovascular morbidity and mortality. Cardiovasc Drugs Ther 1994;8 Suppl 3:549–56. 7841088 10.1007/BF00877223

[10] Maron BJ, Ommen SR, Semsarian C, Spirito P, Olivotto I, Maron MS. Hypertrophic cardiomyopathy: present and future, with translation into contemporary cardiovascular medicine. J Am Coll Cardiol 2014;64:83–99. 24998133 10.1016/j.jacc.2014.05.003

[11] Olivotto I, Cecchi F, Poggesi C, Yacoub MH. Patterns of disease progression in hypertrophic cardiomyopathy: an individualized approach to clinical staging. Circ Heart Fail 2012;5:535–46. 22811549 10.1161/CIRCHEARTFAILURE.112.967026

[12] Marsiglia JD, Pereira AC. Hypertrophic cardiomyopathy: how do mutations lead to disease? Arq Bras Cardiol 2014;102:295–304. 24714796 10.5935/abc.20140022 PMC3987320

[13] McNally EM, Barefield DY, Puckelwartz MJ. The genetic landscape of cardiomyopathy and its role in heart failure. Cell Metab 2015;21:174–82. 25651172 10.1016/j.cmet.2015.01.013 PMC4331062

[14] Carrier L, Mearini G, Stathopoulou K, Cuello F. Cardiac myosin-binding protein C (MYBPC3) in cardiac pathophysiology. Gene 2015;573:188–97. 26358504 10.1016/j.gene.2015.09.008 PMC6660134

[15] Mamidi R, Li J, Doh CY, Verma S, Stelzer JE. Impact of the myosin modulator mavacamten on force generation and cross-bridge behavior in a murine model of hypercontractility. J Am Heart Assoc 2018;7:e009627. 30371160 10.1161/JAHA.118.009627 PMC6201428

[16] Mamidi R, Li J, Gresham KS, Dose-dependent effects of the myosin activator omecamtiv mecarbil on cross-bridge behavior and force generation in failing human myocardium. Circ Heart Fail 2017;10:e004257. 29030372 10.1161/CIRCHEARTFAILURE.117.004257 PMC5685665

[17] Harris SP, Belknap B, Van Sciver RE, White HD, Galkin VE. C0 and C1 N-terminal Ig domains of myosin binding protein C exert different effects on thin filament activation. Proc Natl Acad Sci USA 2016;113:1558–63. 26831109 10.1073/pnas.1518891113 PMC4760775

[18] Dijk SJ, Bezold KL, Harris SP. Earning stripes: myosin binding protein-C interactions with actin. Pflugers Arch 2014;466:445–50. 24442149 10.1007/s00424-013-1432-8 PMC4306388

[19] Moss RL. Cardiac myosin-binding protein C: a protein once at loose ends finds its regulatory groove. Proc Natl Acad Sci USA 2016;113:3133–5. 26966230 10.1073/pnas.1602568113 PMC4812763

[20] Tong CW, Wu X, Liu Y, Phosphoregulation of cardiac inotropy via myosin binding protein-C during increased pacing frequency or β1-adrenergic stimulation. Circ Heart Fail 2015;8:595–604. 25740838 10.1161/CIRCHEARTFAILURE.114.001585 PMC4439328

[21] Rosas PC, Liu Y, Abdalla MI, Phosphorylation of cardiac Myosin-binding protein-C is a critical mediator of diastolic function. Circ Heart Fail 2015;8:582–94. 25740839 10.1161/CIRCHEARTFAILURE.114.001550 PMC4447128

[22] Kubo T, Kitaoka H, Okawa M, Nishinaga M, YL. Hypertrophic cardiomyopathy in the elderly. Geriatr Gerontol Int 2010;10:9–16. 20102377 10.1111/j.1447-0594.2009.00572.x

[23] Baxi AJ, Restrepo CS, Vargas D, Marmol-Velez A, Ocazionez D, Murillo H. Hypertrophic cardiomyopathy from A to Z: genetics, pathophysiology, imaging, and management. Radiographics 2016;36:335–54. 26963450 10.1148/rg.2016150137

[24] Maron BJ, Gardin JM, Flack JM, Gidding SS, Kurosaki TT, Bild DE. Prevalence of hypertrophic cardiomyopathy in a general population of young adults. Circulation 1995;92:785–9. 7641357 10.1161/01.cir.92.4.785

[25] Elliott PM, Anastasakis A, Borger MA, ; Authors/Task Force members. 2014 ESC Guidelines on diagnosis and management of hypertrophic cardiomyopathy: the task force for the diagnosis and management of hypertrophic cardiomyopathy of the European Society of Cardiology (ESC). Eur Heart J 2014;35:2733–79. 25173338 10.1093/eurheartj/ehu284

[26] Bogaert J, Olivotto I. MR Imaging in Hypertrophic cardiomyopathy: from magnet to bedside. Radiology 2014;273:329–48. 25340269 10.1148/radiol.14131626

[27] Soler R, Méndez C, Rodríguez E, Barriales R, Ochoa JP, Monserrat L. Phenotypes of hypertrophic cardiomyopathy. An illustrative review of MRI findings. Insights Imaging 2018;9:1007–20. 30350182 10.1007/s13244-018-0656-8 PMC6269344

[28] Ommen SR, Mital S, Burke MA, 2020 AHA/ACC Guideline for the diagnosis and treatment of patients with hypertrophic cardiomyopathy: executive summary: a report of the American College of Cardiology/American Heart Association Joint Committee on clinical practice guidelines. J Am Coll Cardiol 2020;76:3022–55. 33229115 10.1016/j.jacc.2020.08.044

[29] Maron BJ, Wolfson JK, Epstein SE, Roberts WC. Intramural ("small vessel") coronary artery disease in hypertrophic cardiomyopathy. J Am Coll Cardiol 1986;8:545–57. 3745699 10.1016/s0735-1097(86)80181-4

[30] der Velden J, Stienen GJM. Cardiac disorders and pathophysiology of sarcomeric proteins. Physiol Rev 2019;99:381–426. 30379622 10.1152/physrev.00040.2017

[31] Tian T, Liu Y, Zhou X, Song L. Progress in the molecular genetics of hypertrophic cardiomyopathy: a mini-review. Gerontology 2013;59:199–205. 23363806 10.1159/000346146

[32] Tsao CW, Aday AW, Almarzooq ZI, Heart disease and stroke statistics-2022 update: a report from the American Heart Association. Circulation 2022;145:e153–639. 35078371 10.1161/CIR.0000000000001052

[33] Hershberger RE, Givertz MM, Ho CY, Genetic evaluation of cardiomyopathy-a heart failure society of America Practice Guideline. J Card Fail 2018;24:281–302. 29567486 10.1016/j.cardfail.2018.03.004 PMC9903357

[34] Ho CY, Day SM, Ashley EA, Genotype and lifetime burden of disease in hypertrophic cardiomyopathy: insights from the sarcomeric human cardiomyopathy registry (SHaRe). Circulation 2018;138:1387–98. 30297972 10.1161/CIRCULATIONAHA.117.033200 PMC6170149

[35] Glazier AA, Thompson A, Day SM. Allelic imbalance and haploinsufficiency in MYBPC3-linked hypertrophic cardiomyopathy. Pflugers Arch 2019;471:781–93. 30456444 10.1007/s00424-018-2226-9 PMC6476680

[36] Walsh R, Thomson KL, Ware JS, Reassessment of Mendelian gene pathogenicity using 7,855 cardiomyopathy cases and 60,706 reference samples. Genet Med 2017;19:192–203. 27532257 10.1038/gim.2016.90 PMC5116235

[37] Alfares AA, Kelly MA, McDermott G, Results of clinical genetic testing of 2,912 probands with hypertrophic cardiomyopathy: expanded panels offer limited additional sensitivity. Genet Med 2015;17:880–8. 25611685 10.1038/gim.2014.205

[38] Haas J, Frese KS, Peil B, Atlas of the clinical genetics of human dilated cardiomyopathy. Eur Heart J 2015;36:1123–35a. 25163546 10.1093/eurheartj/ehu301

[39] Pérez-Sánchez I, Romero-Puche AJ, García-Molina Sáez E, Factors influencing the phenotypic expression of hypertrophic cardiomyopathy in genetic carriers. Rev Esp Cardiol 2018;71:146–54. 28687478 10.1016/j.rec.2017.06.002

[40] Maron BJ, Casey SA, Hauser RG, Aeppli DM. Clinical course of hypertrophic cardiomyopathy with survival to advanced age. J Am Coll Cardiol 2003;42:882–8. 12957437 10.1016/s0735-1097(03)00855-6

[41] Minhas AMK, Wyand RA, Ariss RW, Demographic and regional trends of hypertrophic cardiomyopathy-related mortality in the United States, 1999 to 2019. Circ Heart Fail 2022;15:e009292. 36126142 10.1161/CIRCHEARTFAILURE.121.009292

[42] Olivotto I, Maron MS, Adabag AS, Gender-related differences in the clinical presentation and outcome of hypertrophic cardiomyopathy. J Am Coll Cardiol 2005;46:480–7. 16053962 10.1016/j.jacc.2005.04.043

[43] Rowin EJ, Maron MS, Wells S, Patel PP, Koethe BC, Maron BJ. Impact of sex on clinical course and survival in the contemporary treatment era for hypertrophic cardiomyopathy. J Am Heart Assoc 2019;8:e012041. 31663408 10.1161/JAHA.119.012041 PMC6898820

[44] Preveden A, Golubovic M, Bjelobrk M, Gender related differences in the clinical presentation of hypertrophic cardiomyopathy-an analysis from the silicofcm database. Medicina 2022;58:314. 35208637 10.3390/medicina58020314 PMC8879033

[45] Wang Y, Wang J, Zou Y, Female sex is associated with worse prognosis in patients with hypertrophic cardiomyopathy in China. PLoS One 2014;9:e102969. 25047602 10.1371/journal.pone.0102969 PMC4105411

[46] Butters A, Lakdawala NK, Ingles J. Sex differences in hypertrophic cardiomyopathy: interaction with genetics and environment. Curr Heart Fail Rep 2021;18:264–73. 34478112 10.1007/s11897-021-00526-x PMC8484093

[47] Binder J, Ommen SR, Gersh BJ, Echocardiography-guided genetic testing in hypertrophic cardiomyopathy: septal morphological features predict the presence of myofilament mutations. Mayo Clin Proc 2006;81:459–67. 16610565 10.4065/81.4.459

[48] Girolami F, Olivotto I, Passerini I, A molecular screening strategy based on beta-myosin heavy chain, cardiac myosin binding protein C and troponin T genes in Italian patients with hypertrophic cardiomyopathy. J Cardiovasc Med 2006;7:601–7. 10.2459/01.JCM.0000237908.26377.d6 16858239

[49] Marian AJ. Molecular genetic basis of hypertrophic cardiomyopathy. Circ Res 2021;128:1533–53. 33983830 10.1161/CIRCRESAHA.121.318346 PMC8127615

[50] Vakka A, Warren JS, Drosatos K. Cardiovascular aging: from cellular and molecular changes to therapeutic interventions. J Cardiovasc Aging 2023;3:23. 37274126 10.20517/jca.2023.09 PMC10238104

[51] Gude NA, Broughton KM, Firouzi F, Sussman MA. Cardiac ageing: extrinsic and intrinsic factors in cellular renewal and senescence. Nat Rev Cardiol 2018;15:523–42. 30054574 10.1038/s41569-018-0061-5

[52] Dong Y, Xu S, Liu J, Non-coding RNA-linked epigenetic regulation in cardiac hypertrophy. Int J Biol Sci 2018;14:1133–41. 29989099 10.7150/ijbs.26215 PMC6036733

[53] Popa-Fotea NM, Micheu MM, Bataila V, Exploring the continuum of hypertrophic cardiomyopathy-from DNA to clinical expression. Medicina 2019;55:299. 31234582 10.3390/medicina55060299 PMC6630598

[54] Kuno A, Hori YS, Hosoda R, Resveratrol improves cardiomyopathy in dystrophin-deficient mice through SIRT1 protein-mediated modulation of p300 protein. J Biol Chem 2013;288:5963–72. 23297412 10.1074/jbc.M112.392050 PMC3581415

[55] Eom GH, Kook H. Role of histone deacetylase 2 and its posttranslational modifications in cardiac hypertrophy. BMB Rep 2015;48:131–8. 25388210 10.5483/BMBRep.2015.48.3.242 PMC4453031

[56] Vaquero A, Scher M, Lee D, Erdjument-Bromage H, Tempst P, Reinberg D. Human SirT1 interacts with histone H1 and promotes formation of facultative heterochromatin. Mol Cell 2004;16:93–105. 15469825 10.1016/j.molcel.2004.08.031

[57] Cheung P, Vallania F, Warsinske HC, Single-cell chromatin modification profiling reveals increased epigenetic variations with aging. Cell 2018;173:1385–97.e14. 29706550 10.1016/j.cell.2018.03.079 PMC5984186

[58] Sun D, Luo M, Jeong M, Epigenomic profiling of young and aged HSCs reveals concerted changes during aging that reinforce self-renewal. Cell Stem Cell 2014;14:673–88. 24792119 10.1016/j.stem.2014.03.002 PMC4070311

[59] Bell CG, Lowe R, Adams PD, DNA methylation aging clocks: challenges and recommendations. Genome Biol 2019;20:249. 31767039 10.1186/s13059-019-1824-y PMC6876109

[60] Field AE, Robertson NA, Wang T, Havas A, Ideker T, Adams PD. DNA methylation clocks in aging: categories, causes, and consequences. Mol Cell 2018;71:882–95. 30241605 10.1016/j.molcel.2018.08.008 PMC6520108

[61] Koohy H, Bolland DJ, Matheson LS, Genome organization and chromatin analysis identify transcriptional downregulation of insulin-like growth factor signaling as a hallmark of aging in developing B cells. Genome Biol 2018;19:126. 30180872 10.1186/s13059-018-1489-y PMC6124017

[62] Inuzuka Y, Okuda J, Kawashima T, Suppression of phosphoinositide 3-kinase prevents cardiac aging in mice. Circulation 2009;120:1695–703. 19822807 10.1161/CIRCULATIONAHA.109.871137

[63] Costantino S, Paneni F, Cosentino F. Ageing, metabolism and cardiovascular disease. J Physiol 2016;594:2061–73. 26391109 10.1113/JP270538 PMC4933114

[64] Lakatta EG. Arterial and cardiac aging: major shareholders in cardiovascular disease enterprises: Part III: cellular and molecular clues to heart and arterial aging. Circulation 2003;107:490–7. 12551876 10.1161/01.cir.0000048894.99865.02

[65] Gerdes Gyuricza I, Chick JM, Keele GR, Genome-wide transcript and protein analysis highlights the role of protein homeostasis in the aging mouse heart. Genome Res 2022;32:838–52. 35277432 10.1101/gr.275672.121 PMC9104701

[66] Shioi T, Inuzuka Y. Aging as a substrate of heart failure. J Cardiol 2012;60:423–8. 23068289 10.1016/j.jjcc.2012.07.015

[67] Gupta MK, Robbins J. Making the connections: autophagy and post-translational modifications in cardiomyocytes. Autophagy 2016;12:2252–3. 27573291 10.1080/15548627.2016.1215384 PMC5103357

[68] Sharifi-Sanjani M, Oyster NM, Tichy ED, Cardiomyocyte-specific telomere shortening is a distinct signature of heart failure in humans. J Am Heart Assoc 2017;6:e005086. 28882819 10.1161/JAHA.116.005086 PMC5634248

[69] Chang ACY, Chang ACH, Kirillova A, Telomere shortening is a hallmark of genetic cardiomyopathies. Proc Natl Acad Sci USA 2018;115:9276–81. 30150400 10.1073/pnas.1714538115 PMC6140486

[70] Nakada Y, Nhi Nguyen NU, Xiao F, DNA Damage response mediates pressure overload-induced cardiomyocyte hypertrophy. Circulation 2019;139:1237–9. 30802166 10.1161/CIRCULATIONAHA.118.034822 PMC6467068

[71] Wu L, Sowers JR, Zhang Y, Ren J. Targeting DNA damage response in cardiovascular diseases: from pathophysiology to therapeutic implications. Cardiovasc Res 2023;119:691–709. 35576480 10.1093/cvr/cvac080

[72] Aman Y, Schmauck-Medina T, Hansen M, Autophagy in healthy aging and disease. Nat Aging 2021;1:634–50. 34901876 10.1038/s43587-021-00098-4 PMC8659158

[73] Nakai A, Yamaguchi O, Takeda T, The role of autophagy in cardiomyocytes in the basal state and in response to hemodynamic stress. Nat Med 2007;13:619–24. 17450150 10.1038/nm1574

[74] Morimoto S Sarcomeric proteins and inherited cardiomyopathies. Cardiovasc Res 2008;77:659–66. 18056765 10.1093/cvr/cvm084

[75] Stenson PD, Mort M, Ball EV, Shaw K, Phillips A, Cooper DN. The human gene mutation database: building a comprehensive mutation repository for clinical and molecular genetics, diagnostic testing and personalized genomic medicine. Hum Genet 2014;133:1–9. 24077912 10.1007/s00439-013-1358-4 PMC3898141

[76] Viswanathan SK, Sanders HK, McNamara JW, Hypertrophic cardiomyopathy clinical phenotype is independent of gene mutation and mutation dosage. PLoS One 2017;12:e0187948. 29121657 10.1371/journal.pone.0187948 PMC5679632

[77] Fourey D, Care M, Siminovitch KA, Prevalence and clinical implication of double mutations in hypertrophic cardiomyopathy: revisiting the gene-dose effect. Circ Cardiovasc Genet 2017;10:e001685. 28420666 10.1161/CIRCGENETICS.116.001685

[78] Gruen M, Prinz H, Gautel M. cAPK-phosphorylation controls the interaction of the regulatory domain of cardiac myosin binding protein C with myosin-S2 in an on-off fashion. FEBS Lett 1999;453:254–9. 10405155 10.1016/s0014-5793(99)00727-9

[79] Pfuhl M, Gautel M. Structure, interactions and function of the N-terminus of cardiac myosin binding protein C (MyBP-C): who does what, with what, and to whom? J Muscle Res Cell Motil 2012;33:83–94. 22527637 10.1007/s10974-012-9291-z

[80] Witt CC, Gerull B, Davies MJ, Centner T, Linke WA, Thierfelder L. Hypercontractile properties of cardiac muscle fibers in a knock-in mouse model of cardiac myosin-binding protein-C. J Biol Chem 2001;276:5353–9. 11096095 10.1074/jbc.M008691200

[81] Moos C, Offer G, Starr R, Bennett P. Interaction of C-protein with myosin, myosin rod and light meromyosin. J Mol Biol 1975;97:1–9. 1100851 10.1016/s0022-2836(75)80017-9

[82] Freiburg A, Gautel M. A molecular map of the interactions between titin and myosin-binding protein C. Implications for sarcomeric assembly in familial hypertrophic cardiomyopathy. Eur J Biochem 1996;235:317–23. 8631348 10.1111/j.1432-1033.1996.00317.x

[83] James J, Martin L, Krenz M, Forced expression of alpha-myosin heavy chain in the rabbit ventricle results in cardioprotection under cardiomyopathic conditions. Circulation 2005;111:2339–46. 15867177 10.1161/01.CIR.0000164233.09448.B1 PMC1314981

[84] Sadayappan S, Osinska H, Klevitsky R, Cardiac myosin binding protein C phosphorylation is cardioprotective. Proc Natl Acad Sci USA 2006;103:16918–23. 17075052 10.1073/pnas.0607069103 PMC1636554

[85] Barefield D, Sadayappan S. Phosphorylation and function of cardiac myosin binding protein-C in health and disease. J Mol Cell Cardiol 2010;48:866–75. 19962384 10.1016/j.yjmcc.2009.11.014 PMC6800196

[86] Sadayappan S, Gulick J, Klevitsky R, Cardiac myosin binding protein-C phosphorylation in a β-myosin heavy chain background. Circulation 2009;119:1253–62. 19237661 10.1161/CIRCULATIONAHA.108.798983 PMC2656413

[87] Moss RL, Fitzsimons DP, Ralphe JC. Cardiac MyBP-C regulates the rate and force of contraction in mammalian myocardium. Circ Res 2015;116:183–92. 25552695 10.1161/CIRCRESAHA.116.300561 PMC4283578

[88] Desai DA, Rao VJ, Jegga AG, Dhandapany PS, Sadayappan S. Heterogeneous distribution of genetic mutations in myosin binding protein-C paralogs. Front Genet 2022;13:896117. 35832193 10.3389/fgene.2022.896117 PMC9272480

[89] Carrier L Targeting the population for gene therapy with MYBPC3. J Mol Cell Cardiol 2021;150:101–8. 33049255 10.1016/j.yjmcc.2020.10.003

[90] Dhandapany PS, Sadayappan S, Xue Y, A common MYBPC3 (cardiac myosin binding protein C) variant associated with cardiomyopathies in South Asia. Nat Genet 2009;41:187–91. 19151713 10.1038/ng.309 PMC2697598

[91] Jääskeläinen P, Miettinen R, Kärkkäinen P, Toivonen L, Laakso M, Kuusisto J. Genetics of hypertrophic cardiomyopathy in eastern Finland: few founder mutations with benign or intermediary phenotypes. Ann Med 2004;36:23–32. 15000344 10.1080/07853890310017161

[92] Kubo T, Kitaoka H, Okawa M, Lifelong left ventricular remodeling of hypertrophic cardiomyopathy caused by a founder frameshift deletion mutation in the cardiac Myosin-binding protein C gene among Japanese. J Am Coll Cardiol 2005;46:1737–43. 16256878 10.1016/j.jacc.2005.05.087

[93] Michels M, Soliman OI, Kofflard MJ, Diastolic abnormalities as the first feature of hypertrophic cardiomyopathy in Dutch myosin-binding protein C founder mutations. JACC Cardiovasc Imaging 2009;2:58–64. 19356534 10.1016/j.jcmg.2008.08.003

[94] Niimura H, Bachinski LL, Sangwatanaroj S, Mutations in the gene for cardiac myosin-binding protein C and late-onset familial hypertrophic cardiomyopathy. N Engl J Med 1998;338:1248–57. 9562578 10.1056/NEJM199804303381802

[95] Suay-Corredera C, Pricolo MR, Herrero-Galán E, Protein haploinsufficiency drivers identify MYBPC3 variants that cause hypertrophic cardiomyopathy. J Biol Chem 2021;297:100854. 34097875 10.1016/j.jbc.2021.100854 PMC8260873

[96] Mazzarotto F, Olivotto I, Boschi B, Contemporary insights into the genetics of hypertrophic cardiomyopathy: toward a new era in clinical testing? J Am Heart Assoc 2020;9:e015473. 32306808 10.1161/JAHA.119.015473 PMC7428545

[97] Torrado M, Maneiro E, Lamounier Junior A, Identification of an elusive spliceogenic MYBPC3 variant in an otherwise genotype-negative hypertrophic cardiomyopathy pedigree. Sci Rep 2022;12:7284. 35508642 10.1038/s41598-022-11159-y PMC9068804

[98] Lopes LR, Barbosa P, Torrado M, Cryptic splice-altering variants in MYBPC3 are a prevalent cause of hypertrophic cardiomyopathy. Circ Genom Precis Med 2020;13:e002905. 32396390 10.1161/CIRCGEN.120.002905

[99] Niimura H, Patton KK, McKenna WJ, Sarcomere protein gene mutations in hypertrophic cardiomyopathy of the elderly. Circulation 2002;105:446–51. 11815426 10.1161/hc0402.102990

[100] Maron BJ, Niimura H, Casey SA, Development of left ventricular hypertrophy in adults in hypertrophic cardiomyopathy caused by cardiac myosin-binding protein C gene mutations. J Am Coll Cardiol 2001;38:315–21. 11499718 10.1016/s0735-1097(01)01386-9

[101] Hirota T, Kitaoka H, Kubo T, Okawa M, Furuno T, YL. Morphologic characteristics of hypertrophic cardiomyopathy of the elderly with cardiac myosin-binding protein C gene mutations. Circ J 2006;70:875–9. 16799241 10.1253/circj.70.875

[102] McConnell BK, Fatkin D, Semsarian C, Comparison of two murine models of familial hypertrophic cardiomyopathy. Circ Res 2001;88:383–9. 11230104 10.1161/01.res.88.4.383

[103] Sato N, Kawakami T, Nakayama A, Suzuki H, Kasahara H, Obinata T. A novel variant of cardiac myosin-binding protein-C that is unable to assemble into sarcomeres is expressed in the aged mouse atrium. Mol Biol Cell 2003;14:3180–91. 12925755 10.1091/mbc.E02-10-0685 PMC181559

[104] Vignier N, Schlossarek S, Fraysse B, Nonsense-mediated mRNA decay and ubiquitin-proteasome system regulate cardiac myosin-binding protein C mutant levels in cardiomyopathic mice. Circ Res 2009;105:239–48. 19590044 10.1161/CIRCRESAHA.109.201251

[105] van Dijk SJ, Dooijes D, dos Remedios C, Cardiac myosin-binding protein C mutations and hypertrophic cardiomyopathy: haploinsufficiency, deranged phosphorylation, and cardiomyocyte dysfunction. Circulation 2009;119:1473–83. 19273718 10.1161/CIRCULATIONAHA.108.838672

[106] Nakamura M, Sadoshima J. Mechanisms of physiological and pathological cardiac hypertrophy. Nat Rev Cardiol 2018;15:387–407. 29674714 10.1038/s41569-018-0007-y

[107] Ren X, Hensley N, Brady MB, Gao WD. The genetic and molecular bases for hypertrophic cardiomyopathy: the role for calcium sensitization. J Cardiothorac Vasc Anesth 2018;32:478–87. 29203298 10.1053/j.jvca.2017.05.035

[108] Parbhudayal RY, Garra AR, Götte MJW, Variable cardiac myosin binding protein-C expression in the myofilaments due to MYBPC3 mutations in hypertrophic cardiomyopathy. J Mol Cell Cardiol 2018;123:59–63. 30170119 10.1016/j.yjmcc.2018.08.023

[109] Lin B, Govindan S, Lee K, Cardiac myosin binding protein-C plays no regulatory role in skeletal muscle structure and function. PLoS One 2013;8:e69671. 23936073 10.1371/journal.pone.0069671 PMC3729691

[110] Akazawa H, Komuro I. Roles of cardiac transcription factors in cardiac hypertrophy. Circ Res 2003;92:1079–88. 12775656 10.1161/01.RES.0000072977.86706.23

[111] Charron F, Nemer M. GATA transcription factors and cardiac development. Semin Cell Dev Biol 1999;10:85–91. 10355032 10.1006/scdb.1998.0281

[112] Dodou E, Xu SM, Black BL. mef2c is activated directly by myogenic basic helix-loop-helix proteins during skeletal muscle development in vivo. Mech Dev 2003;120:1021–32. 14550531 10.1016/s0925-4773(03)00178-3

[113] Farrell E, Armstrong AE, Grimes AC, Naya FJ, de Lange WJ, Ralphe JC. Transcriptome analysis of cardiac hypertrophic growth in MYBPC3-null mice suggests early responders in hypertrophic remodeling. Front Physiol 2018;9:1442. 30410445 10.3389/fphys.2018.01442 PMC6210548

[114] Pei J, Schuldt M, Nagyova E, Multi-omics integration identifies key upstream regulators of pathomechanisms in hypertrophic cardiomyopathy due to truncating MYBPC3 mutations. Clin Epigenetics 2021;13:61. 33757590 10.1186/s13148-021-01043-3 PMC7989210

[115] Meurs KM, Kuan M. Differential methylation of CpG sites in two isoforms of myosin binding protein C, an important hypertrophic cardiomyopathy gene. Environ Mol Mutagen 2011;52:161–4. 20740642 10.1002/em.20596

[116] Keene JD. RNA regulons: coordination of post-transcriptional events. Nat Rev Genet 2007;8:533–43. 17572691 10.1038/nrg2111

[117] Mata J, Marguerat S, Bähler J. Post-transcriptional control of gene expression: a genome-wide perspective. Trends Biochem Sci 2005;30:506–14. 16054366 10.1016/j.tibs.2005.07.005

[118] Gehring NH, Roignant JY. Anything but ordinary - emerging splicing mechanisms in eukaryotic gene regulation. Trends Genet 2021;37:355–72. 33203572 10.1016/j.tig.2020.10.008

[119] Sciabica KS, Hertel KJ. The splicing regulators Tra and Tra2 are unusually potent activators of pre-mRNA splicing. Nucleic Acids Res 2006;34:6612–20. 17135210 10.1093/nar/gkl984 PMC1747189

[120] Han J, Cooper TA. Identification of CELF splicing activation and repression domains in vivo. Nucleic Acids Res 2005;33:2769–80. 15894795 10.1093/nar/gki561 PMC1126903

[121] Hasimbegovic E, Schweiger V, Kastner N, Alternative splicing in cardiovascular disease-a survey of recent findings. Genes 2021;12:1457. 34573439 10.3390/genes12091457 PMC8469243

[122] Beqqali A. Alternative splicing in cardiomyopathy. Biophys Rev 2018;10:1061–71. 30051286 10.1007/s12551-018-0439-y PMC6082314

[123] Dai J, Li Z, Huang W, RBM20 is a candidate gene for hypertrophic cardiomyopathy. Can J Cardiol 2021;37:1751–9. 34333030 10.1016/j.cjca.2021.07.014

[124] Guo W, Schafer S, Greaser ML, RBM20, a gene for hereditary cardiomyopathy, regulates titin splicing. Nat Med 2012;18:766–73. 22466703 10.1038/nm.2693 PMC3569865

[125] Akerberg AA, Trembley M, Butty V, RBPMS2 is a myocardial-enriched splicing regulator required for cardiac function. Circ Res 2022;131:980–1000. 36367103 10.1161/CIRCRESAHA.122.321728 PMC9770155

[126] Lu SH, Lee KZ, Hsu PW, Alternative splicing mediated by RNA-binding protein RBM24 facilitates cardiac myofibrillogenesis in a differentiation stage-specific manner. Circ Res 2022;130:112–29. 34816743 10.1161/CIRCRESAHA.121.320080

[127] Bhuvanagiri M, Schlitter AM, Hentze MW, Kulozik AE. NMD: RNA biology meets human genetic medicine. Biochem J 2010;430:365–77. 20795950 10.1042/BJ20100699

[128] Helms AS, Thompson AD, Glazier AA, Spatial and functional distribution of MYBPC3 pathogenic variants and clinical outcomes in patients with hypertrophic cardiomyopathy. Circ Genom Precis Med 2020;13:396–405. 32841044 10.1161/CIRCGEN.120.002929 PMC7676622

[129] Herron TJ, Rostkova E, Kunst G, Chaturvedi R, Gautel M, Kentish JC. Activation of myocardial contraction by the N-terminal domains of myosin binding protein-C. Circ Res 2006;98:1290–8. 16614305 10.1161/01.RES.0000222059.54917.ef

[130] Govindan S, Sarkey J, Ji X, Pathogenic properties of the N-terminal region of cardiac myosin binding protein-C in vitro. J Muscle Res Cell Motil 2012;33:17–30. 22527638 10.1007/s10974-012-9292-y PMC3368277

[131] Yang Q, Sanbe A, Osinska H, Hewett TE, Klevitsky R, Robbins J. A mouse model of myosin binding protein C human familial hypertrophic cardiomyopathy. J Clin Invest 1998;102:1292–300. 9769321 10.1172/JCI3880 PMC508976

[132] Yang Q, Sanbe A, Osinska H, Hewett TE, Klevitsky R, Robbins J. In vivo modeling of myosin binding protein C familial hypertrophic cardiomyopathy. Circ Res 1999;85:841–7. 10532952 10.1161/01.res.85.9.841

[133] Kuster DWD, Lynch TL, Barefield DY, Altered C10 domain in cardiac myosin binding protein-C results in hypertrophic cardiomyopathy. Cardiovasc Res 2019;115:1986–97. 31050699 10.1093/cvr/cvz111 PMC6872972

[134] Razzaque MA, Gupta M, Osinska H, Gulick J, Blaxall BC, Robbins J. An endogenously produced fragment of cardiac myosin-binding protein C is pathogenic and can lead to heart failure. Circ Res 2013;113:553–61. 23852539 10.1161/CIRCRESAHA.113.301225 PMC3835189

[135] Li J, Mamidi R, Doh CY, AAV9 gene transfer of cMyBPC N-terminal domains ameliorates cardiomyopathy in cMyBPC-deficient mice. JCI Insight 2020;5:130182. 32750038 10.1172/jci.insight.130182 PMC7526450

[136] Rottbauer W, Gautel M, Zehelein J, Novel splice donor site mutation in the cardiac myosin-binding protein-C gene in familial hypertrophic cardiomyopathy. Characterization of cardiac transcript and protein. J Clin Invest 1997;100:475–82. 9218526 10.1172/JCI119555 PMC508212

[137] Moolman JA, Reith S, Uhl K, A newly created splice donor site in exon 25 of the MyBP-C gene is responsible for inherited hypertrophic cardiomyopathy with incomplete disease penetrance. Circulation 2000;101:1396–402. 10736283 10.1161/01.cir.101.12.1396

[138] Marston S, Copeland O, Jacques A, Evidence from human myectomy samples that MYBPC3 mutations cause hypertrophic cardiomyopathy through haploinsufficiency. Circ Res 2009;105:219–22. 19574547 10.1161/CIRCRESAHA.109.202440

[139] Seeger T, Shrestha R, Lam CK, A Premature termination codon mutation in MYBPC3 causes hypertrophic cardiomyopathy via chronic activation of nonsense-mediated decay. Circulation 2019;139:799–811. 30586709 10.1161/CIRCULATIONAHA.118.034624 PMC6443405

[140] Burkart V, Kowalski K, Disch A, Nonsense mediated decay factor UPF3B is associated with cMyBP-C haploinsufficiency in hypertrophic cardiomyopathy patients. J Mol Cell Cardiol 2023;185:26–37. 37797718 10.1016/j.yjmcc.2023.09.008

[141] Iwakawa HO, Tomari Y. Life of RISC: formation, action, and degradation of RNA-induced silencing complex. Mol Cell 2022;82:30–43. 34942118 10.1016/j.molcel.2021.11.026

[142] Kuster DW, Mulders J, Ten Cate FJ, MicroRNA transcriptome profiling in cardiac tissue of hypertrophic cardiomyopathy patients with MYBPC3 mutations. J Mol Cell Cardiol 2013;65:59–66. 24083979 10.1016/j.yjmcc.2013.09.012

[143] Lin LR, Hu XQ, Lu LH, MicroRNA expression profiles in familial hypertrophic cardiomyopathy with myosin-binding protein C3 (MYBPC3) gene mutations. BMC Cardiovasc Disord 2022;22:278. 35717150 10.1186/s12872-022-02714-6 PMC9206743

[144] Walsh CT, Garneau-Tsodikova S, Gatto GJ Jr. Protein posttranslational modifications: the chemistry of proteome diversifications. Angew Chem Int Ed 2005;44:7342–72. 10.1002/anie.200501023 16267872

[145] Yan K, Wang K, Li P. The role of post-translational modifications in cardiac hypertrophy. J Cell Mol Med 2019;23:3795–807. 30950211 10.1111/jcmm.14330 PMC6533522

[146] Sadayappan S, Gulick J, Osinska H, Cardiac myosin-binding protein-C phosphorylation and cardiac function. Circ Res 2005;97:1156–63. 16224063 10.1161/01.RES.0000190605.79013.4d PMC1343494

[147] El-Armouche A, Pohlmann L, Schlossarek S, Decreased phosphorylation levels of cardiac myosin-binding protein-C in human and experimental heart failure. J Mol Cell Cardiol 2007;43:223–9. 17560599 10.1016/j.yjmcc.2007.05.003

[148] Copeland ON, Sadayappan S, Messer AE, Steinen GJM, van der Velden J, Marston SB. Analysis of cardiac myosin binding protein-C phosphorylation in human heart muscle. J Mol Cell Cardiol 2010;49:1003–11. 20850451 10.1016/j.yjmcc.2010.09.007

[149] Stelzer JE, Patel JR, Moss RL. Protein kinase A-mediated acceleration of the stretch activation response in murine skinned myocardium is eliminated by ablation of cMyBP-C. Circ Res 2006;99:884–90. 16973906 10.1161/01.RES.0000245191.34690.66

[150] Stelzer JE, Patel JR, Walker JW, Moss RL. Differential roles of cardiac myosin-binding protein C and cardiac troponin I in the myofibrillar force responses to protein kinase A phosphorylation. Circ Res 2007;101:503–11. 17641226 10.1161/CIRCRESAHA.107.153650

[151] Lynch TL 4th, Kumar M, McNamara JW, Amino terminus of cardiac myosin binding protein-C regulates cardiac contractility. J Mol Cell Cardiol 2021;156:33–44. 33781820 10.1016/j.yjmcc.2021.03.009 PMC8217138

[152] Lim MS, Sutherland C, Walsh MP. Phosphorylation of bovine cardiac C-protein by protein kinase C. Biochem Biophys Res Commun 1985;132:1187–95. 3840998 10.1016/0006-291x(85)91932-1

[153] Venema RC, Kuo JF. Protein kinase C-mediated phosphorylation of troponin I and C-protein in isolated myocardial cells is associated with inhibition of myofibrillar actomyosin MgATPase. J Biol Chem 1993;268:2705–11. 8381412

[154] Mohamed AS, Dignam JD, Schlender KK. Cardiac myosin-binding protein C (MyBP-C): identification of protein kinase A and protein kinase C phosphorylation sites. Arch Biochem Biophys 1998;358:313–9. 9784245 10.1006/abbi.1998.0857

[155] Sadayappan S, Gulick J, Osinska H, A critical function for Ser-282 in cardiac Myosin binding protein-C phosphorylation and cardiac function. Circ Res 2011;109:141–50. 21597010 10.1161/CIRCRESAHA.111.242560 PMC3132348

[156] Tong CW, Gaffin RD, Zawieja DC, Muthuchamy M. Roles of phosphorylation of myosin binding protein-C and troponin I in mouse cardiac muscle twitch dynamics. J Physiol 2004;558:927–41. 15194741 10.1113/jphysiol.2004.062539 PMC1665013

[157] Kooij V, Holewinski RJ, Murphy AM, Van Eyk JE. Characterization of the cardiac myosin binding protein-C phosphoproteome in healthy and failing human hearts. J Mol Cell Cardiol 2013;60:116–20. 23619294 10.1016/j.yjmcc.2013.04.012 PMC3710717

[158] Kuster DW, Sequeira V, Najafi A, GSK3β phosphorylates newly identified site in the proline-alanine-rich region of cardiac myosin-binding protein C and alters cross-bridge cycling kinetics in human: short communication. Circ Res 2013;112:633–9. 23277198 10.1161/CIRCRESAHA.112.275602 PMC3595322

[159] Barefield D, Kumar M, de Tombe PP, Sadayappan S. Contractile dysfunction in a mouse model expressing a heterozygous MYBPC3 mutation associated with hypertrophic cardiomyopathy. Am J Physiol Heart Circ Physiol 2014;306:H807–15. 24464755 10.1152/ajpheart.00913.2013 PMC3949045

[160] Lovelock JD, Monasky MM, Jeong EM, Ranolazine improves cardiac diastolic dysfunction through modulation of myofilament calcium sensitivity. Circ Res 2012;110:841–50. 22343711 10.1161/CIRCRESAHA.111.258251 PMC3314887

[161] Patel BG, Wilder T, Solaro RJ. Novel control of cardiac myofilament response to calcium by S-glutathionylation at specific sites of myosin binding protein C. Front Physiol 2013;4:336. 24312057 10.3389/fphys.2013.00336 PMC3834529

[162] Fert-Bober J, Sokolove J. Proteomics of citrullination in cardiovascular disease. Proteomics Clin Appl 2014;8:522–33. 24946285 10.1002/prca.201400013

[163] Barefield DY, McNamara JW, Lynch TL, Ablation of the calpain-targeted site in cardiac myosin binding protein-C is cardioprotective during ischemia-reperfusion injury. J Mol Cell Cardiol 2019;129:236–46. 30862451 10.1016/j.yjmcc.2019.03.006 PMC7222036

[164] Braten O, Livneh I, Ziv T, Numerous proteins with unique characteristics are degraded by the 26S proteasome following monoubiquitination. Proc Natl Acad Sci USA 2016;113:E4639–47. 27385826 10.1073/pnas.1608644113 PMC4987823

[165] Oh E, Akopian D, Rape M. Principles of ubiquitin-dependent signaling. Annu Rev Cell Dev Biol 2018;34:137–62. 30110556 10.1146/annurev-cellbio-100617-062802

[166] Dikic I. Proteasomal and autophagic degradation systems. Annu Rev Biochem 2017;86:193–224. 28460188 10.1146/annurev-biochem-061516-044908

[167] Park J, Cho J, Song EJ. Ubiquitin-proteasome system (UPS) as a target for anticancer treatment. Arch Pharm Res 2020;43:1144–61. 33165832 10.1007/s12272-020-01281-8 PMC7651821

[168] Pohl C, Dikic I. Cellular quality control by the ubiquitin-proteasome system and autophagy. Science 2019;366:818–22. 31727826 10.1126/science.aax3769

[169] Helms AS, Tang VT, OĽeary TS, Effects of MYBPC3 loss-of-function mutations preceding hypertrophic cardiomyopathy. JCI Insight 2020;5:133782. 31877118 10.1172/jci.insight.133782 PMC7098724

[170] Sarikas A, Carrier L, Schenke C, Impairment of the ubiquitin-proteasome system by truncated cardiac myosin binding protein C mutants. Cardiovasc Res 2005;66:33–44. 15769446 10.1016/j.cardiores.2005.01.004

[171] Mearini G, Schlossarek S, Willis MS, Carrier L. The ubiquitin-proteasome system in cardiac dysfunction. Biochim Biophys Acta 2008;1782:749–63. 18634872 10.1016/j.bbadis.2008.06.009

[172] Mearini G, Gedicke C, Schlossarek S, Atrogin-1 and MuRF1 regulate cardiac MyBP-C levels via different mechanisms. Cardiovasc Res 2010;85:357–66. 19850579 10.1093/cvr/cvp348 PMC4023316

[173] Thottakara T, Friedrich FW, Reischmann S, The E3 ubiquitin ligase Asb2β is downregulated in a mouse model of hypertrophic cardiomyopathy and targets desmin for proteasomal degradation. J Mol Cell Cardiol 2015;87:214–24. 26343497 10.1016/j.yjmcc.2015.08.020

[174] Galluzzi L, Baehrecke EH, Ballabio A, Molecular definitions of autophagy and related processes. EMBO J 2017;36:1811–36. 28596378 10.15252/embj.201796697 PMC5494474

[175] Glazier AA, Hafeez N, Mellacheruvu D, HSC70 is a chaperone for wild-type and mutant cardiac myosin binding protein C. JCI Insight 2018;3:99319. 29875314 10.1172/jci.insight.99319 PMC6124431

[176] Kaushik S, Cuervo AM. The coming of age of chaperone-mediated autophagy. Nat Rev Mol Cell Biol 2018;19:365–81. 29626215 10.1038/s41580-018-0001-6 PMC6399518

[177] Martin TG, Myers VD, Dubey P, Cardiomyocyte contractile impairment in heart failure results from reduced BAG3-mediated sarcomeric protein turnover. Nat Commun 2021;12:2942. 34011988 10.1038/s41467-021-23272-z PMC8134551

[178] Hishiya A, Kitazawa T, Takayama S. BAG3 and Hsc70 interact with actin capping protein CapZ to maintain myofibrillar integrity under mechanical stress. Circ Res 2010;107:1220–31. 20884878 10.1161/CIRCRESAHA.110.225649 PMC2980587

[179] Bhadra M, Howell P, Dutta S, Heintz C, Mair WB. Alternative splicing in aging and longevity. Hum Genet 2020;139:357–69. 31834493 10.1007/s00439-019-02094-6 PMC8176884

[180] Harries LW, Hernandez D, Henley W, Human aging is characterized by focused changes in gene expression and deregulation of alternative splicing. Aging Cell 2011;10:868–78. 21668623 10.1111/j.1474-9726.2011.00726.x PMC3173580

[181] Angarola BL, Anczuków O. Splicing alterations in healthy aging and disease. Wiley Interdiscip Rev RNA 2021;12:e1643. 33565261 10.1002/wrna.1643 PMC8195850

[182] Yao J, Ding D, Li X, Prevalent intron retention fine-tunes gene expression and contributes to cellular senescence. Aging Cell 2020;19:e13276. 33274830 10.1111/acel.13276 PMC7744961

[183] Wang K, Wu D, Zhang H, Comprehensive map of age-associated splicing changes across human tissues and their contributions to age-associated diseases. Sci Rep 2018;8:10929. 30026530 10.1038/s41598-018-29086-2 PMC6053367

[184] Rodríguez SA, Grochová D, McKenna T, Global genome splicing analysis reveals an increased number of alternatively spliced genes with aging. Aging Cell 2016;15:267–78. 26685868 10.1111/acel.12433 PMC4783335

[185] Heintz C, Doktor TK, Lanjuin A, Splicing factor 1 modulates dietary restriction and TORC1 pathway longevity in *C. elegans*. Nature 2017;541:102–6. 27919065 10.1038/nature20789 PMC5361225

[186] Mazin P, Xiong J, Liu X, Widespread splicing changes in human brain development and aging. Mol Syst Biol 2013;9:633. 23340839 10.1038/msb.2012.67 PMC3564255

[187] Pagani F, Zagato L, Vergani C, Casari G, Sidoli A, Baralle FE. Tissue-specific splicing pattern of fibronectin messenger RNA precursor during development and aging in rat. J Cell Biol 1991;113:1223–9. 2040649 10.1083/jcb.113.5.1223 PMC2289010

[188] Debès C, Papadakis A, Grönke S, Ageing-associated changes in transcriptional elongation influence longevity. Nature 2023;616:814–21. 37046086 10.1038/s41586-023-05922-y PMC10132977

[189] Blanco FJ, Bernabéu C. The splicing factor SRSF1 as a marker for endothelial senescence. Front Physiol 2012;3:54. 22470345 10.3389/fphys.2012.00054 PMC3314196

[190] Fregoso OI, Das S, Akerman M, Krainer AR. Splicing-factor oncoprotein SRSF1 stabilizes p53 via RPL5 and induces cellular senescence. Mol Cell 2013;50:56–66. 23478443 10.1016/j.molcel.2013.02.001 PMC3628402

[191] Tang Y, Horikawa I, Ajiro M, Downregulation of splicing factor SRSF3 induces p53β, an alternatively spliced isoform of p53 that promotes cellular senescence. Oncogene 2013;32:2792–8. 22777358 10.1038/onc.2012.288 PMC6503963

[192] Han Y, Wennersten SA, Wright JM, Ludwig RW, Lau E, Lam MPY. Proteogenomics reveals sex-biased aging genes and coordinated splicing in cardiac aging. Am J Physiol Heart Circ Physiol 2022;323:H538–58. 35930447 10.1152/ajpheart.00244.2022 PMC9448281

[193] Kadota Y, Jam FA, Yukiue H, Srsf7 establishes the juvenile transcriptome through age-dependent alternative splicing in mice. iScience 2020;23:100929. 32146325 10.1016/j.isci.2020.100929 PMC7063262

[194] Zhang M, Zhang Y, Xu E, Rbm24, a target of p53, is necessary for proper expression of p53 and heart development. Cell Death Differ 2018;25:1118–30. 29358667 10.1038/s41418-017-0029-8 PMC5988652

[195] Morita H, Komuro I. Heart failure as an aging-related phenotype. Int Heart J 2018;59:6–13. 29332923 10.1536/ihj.17-519

[196] Cheng M, Zhan X, Xu Y, DNA methylation of RNA-binding protein for multiple splicing 2 functions as diagnosis biomarker in gastric cancer pathogenesis and its potential clinical significance. Bioengineered 2022;13:4347–60. 35137653 10.1080/21655979.2022.2032965 PMC8973754

[197] Waldera-Lupa DM, Kalfalah F, Florea AM, Proteome-wide analysis reveals an age-associated cellular phenotype of in situ aged human fibroblasts. Aging 2014;6:856–78. 25411231 10.18632/aging.100698 PMC4247387

[198] Kalfalah F, Sobek S, Bornholz B, Inadequate mito-biogenesis in primary dermal fibroblasts from old humans is associated with impairment of PGC1A-independent stimulation. Exp Gerontol 2014;56:59–68. 24699405 10.1016/j.exger.2014.03.017

[199] Son HG, Seo M, Ham S, RNA surveillance via nonsense-mediated mRNA decay is crucial for longevity in daf-2/insulin/IGF-1 mutant C. elegans. Nat Commun 2017;8:14749. 28276441 10.1038/ncomms14749 PMC5347137

[200] Masse I, Molin L, Mouchiroud L, A novel role for the SMG-1 kinase in lifespan and oxidative stress resistance in Caenorhabditis elegans. PLoS One 2008;3:e3354. 18836529 10.1371/journal.pone.0003354 PMC2556085

[201] Huth M, Santini L, Galimberti E, NMD is required for timely cell fate transitions by fine-tuning gene expression and regulating translation. Genes Dev 2022;36:348–67. 35241478 10.1101/gad.347690.120 PMC8973849

[202] Boehm M, Slack F. A developmental timing microRNA and its target regulate life span in *C. elegans*. Science 2005;310:1954–7. 16373574 10.1126/science.1115596

[203] Hooten N, Abdelmohsen K, Gorospe M, Ejiogu N, Zonderman AB, Evans MK. microRNA expression patterns reveal differential expression of target genes with age. PLoS One 2010;5:e10724. 20505758 10.1371/journal.pone.0010724 PMC2873959

[204] Pincus Z, Smith-Vikos T, Slack FJ. MicroRNA predictors of longevity in Caenorhabditis elegans. PLoS Genet 2011;7:e1002306. 21980307 10.1371/journal.pgen.1002306 PMC3183074

[205] Smith-Vikos T, Slack FJ. MicroRNAs and their roles in aging. J Cell Sci 2012;125:7–17. 22294612 10.1242/jcs.099200 PMC3269020

[206] ElSharawy A, Keller A, Flachsbart F, Genome-wide miRNA signatures of human longevity. Aging Cell 2012;11:607–16. 22533606 10.1111/j.1474-9726.2012.00824.x

[207] Tudurachi BS, Zăvoi A, Leonte A, An update on *MYBPC3* gene mutation in hypertrophic cardiomyopathy. Int J Mol Sci 2023;24:10510. 37445689 10.3390/ijms241310510 PMC10341819

[208] Jung HJ, Suh Y. MicroRNA in aging: from discovery to biology. Curr Genomics 2012;13:548–57. 23633914 10.2174/138920212803251436 PMC3468887

[209] Kinser HE, Pincus Z. MicroRNAs as modulators of longevity and the aging process. Hum Genet 2020;139:291–308. 31297598 10.1007/s00439-019-02046-0 PMC6954352

[210] de Lucia C, Komici K, Borghetti G, microRNA in cardiovascular aging and age-related cardiovascular diseases. Front Med 2017;4:74. 10.3389/fmed.2017.00074 PMC5466994 28660188

[211] Lefkowitz RJ, Rockman HA, Koch WJ. Catecholamines, cardiac beta-adrenergic receptors, and heart failure. Circulation 2000;101:1634–7. 10758041 10.1161/01.cir.101.14.1634

[212] Malik FI, Morgan BP. Cardiac myosin activation part 1: from concept to clinic. J Mol Cell Cardiol 2011;51:454–61. 21616079 10.1016/j.yjmcc.2011.05.006

[213] Malik FI, Hartman JJ, Elias KA, Cardiac myosin activation: a potential therapeutic approach for systolic heart failure. Science 2011;331:1439–43. 21415352 10.1126/science.1200113 PMC4090309

[214] Zhao X, Ho D, Abarzúa P, Inhibition of smooth muscle myosin as a novel therapeutic target for hypertension. J Pharmacol Exp Ther 2011;339:307–12. 21784887 10.1124/jpet.111.182402 PMC3186291

[215] Bergonzo C, Aryal B, Rao VA. Divalent ions as mediators of carbonylation in cardiac myosin binding protein C. J Mol Graph Model 2023;124:108576. 37536231 10.1016/j.jmgm.2023.108576

[216] Rosas PC, Solaro RJ. Implications of S-glutathionylation of sarcomere proteins in cardiac disorders, therapies, and diagnosis. Front Cardiovasc Med 2022;9:1060716. 36762302 10.3389/fcvm.2022.1060716 PMC9902711

[217] Suay-Corredera C, Alegre-Cebollada J. The mechanics of the heart: zooming in on hypertrophic cardiomyopathy and cMyBP-C. FEBS Lett 2022;596:703–46. 35224729 10.1002/1873-3468.14301

[218] Main A, Fuller W, Baillie GS. Post-translational regulation of cardiac myosin binding protein-C: a graphical review. Cell Signal 2020;76:109788. 32976931 10.1016/j.cellsig.2020.109788

[219] Heling LWHJ Geeves MA, Kad NM. MyBP-C: one protein to govern them all. J Muscle Res Cell Motil 2020;41:91–101. 31960266 10.1007/s10974-019-09567-1 PMC7109175

[220] Enns LC, Pettan-Brewer C, Ladiges W. Protein kinase A is a target for aging and the aging heart. Aging 2010;2:238–43. 20448293 10.18632/aging.100138 PMC2881512

[221] Enns LC, Ladiges W. Protein kinase A signaling as an anti-aging target. Ageing Res Rev 2010;9:269–72. 20188216 10.1016/j.arr.2010.02.004

[222] Nattel S. Aging and protein kinase activation: is it the missing link between age and atrial fibrillation? Circ Res 2018;122:799–801. 29700079 10.1161/CIRCRESAHA.118.312786

[223] Wang HY, Bashore TR, Tran ZV, Friedman E. Age-related decreases in lymphocyte protein kinase C activity and translocation are reduced by aerobic fitness. J Gerontol A Biol Sci Med Sci 2000;55:B545–51. 11078088 10.1093/gerona/55.11.b545

[224] Battaini F, Elkabes S, Bergamaschi S, Protein kinase C activity, translocation, and conventional isoforms in aging rat brain. Neurobiol Aging 1995;16:137–48. 7777132 10.1016/0197-4580(94)00154-5

[225] Kane AE, Bisset ES, Keller KM, Ghimire A, Pyle WG, Howlett SE. Age, sex and overall health, measured as frailty, modify myofilament proteins in hearts from naturally aging mice. Sci Rep 2020;10:10052. 32572088 10.1038/s41598-020-66903-z PMC7308399

[226] Yuan C, Sheng Q, Tang H, Li Y, Zeng R, Solaro RJ. Quantitative comparison of sarcomeric phosphoproteomes of neonatal and adult rat hearts. Am J Physiol Heart Circ Physiol 2008;295:H647–56. 18552161 10.1152/ajpheart.00357.2008 PMC2519213

[227] Rosas PC, Warren CM, Creed HA, Trzeciakowski JP, Solaro RJ, Tong CW. Cardiac myosin binding protein-C phosphorylation mitigates age-related cardiac dysfunction: hope for better aging? JACC Basic Transl Sci 2019;4:817–30. 31998850 10.1016/j.jacbts.2019.06.003 PMC6978553

[228] McNamara JW, Li A, Lal S, *MYBPC3* mutations are associated with a reduced super-relaxed state in patients with hypertrophic cardiomyopathy. PLoS One 2017;12:e0180064. 28658286 10.1371/journal.pone.0180064 PMC5489194

[229] Nakayama H, Nishida K, Otsu K. Macromolecular degradation systems and cardiovascular aging. Circ Res 2016;118:1577–92. 27174951 10.1161/CIRCRESAHA.115.307495

[230] Powell SR. The ubiquitin-proteasome system in cardiac physiology and pathology. Am J Physiol Heart Circ Physiol 2006;291:H1–19. 16501026 10.1152/ajpheart.00062.2006

[231] Salcan S, Bongardt S, Monteiro Barbosa D, Elastic titin properties and protein quality control in the aging heart. Biochim Biophys Acta Mol Cell Res 2020;1867:118532. 31421188 10.1016/j.bbamcr.2019.118532

[232] Li F, Zhang L, Craddock J, Aging and dietary restriction effects on ubiquitination, sumoylation, and the proteasome in the heart. Mech Ageing Dev 2008;129:515–21. 18533226 10.1016/j.mad.2008.04.007 PMC2546525

[233] Bulteau AL, Szweda LI, Friguet B. Age-dependent declines in proteasome activity in the heart. Arch Biochem Biophys 2002;397:298–304. 11795886 10.1006/abbi.2001.2663

[234] Sosnowska D, Richardson C, Sonntag WE, Csiszar A, Ungvari Z, Ridgway I. A heart that beats for 500 years: age-related changes in cardiac proteasome activity, oxidative protein damage and expression of heat shock proteins, inflammatory factors, and mitochondrial complexes in Arctica islandica, the longest-living noncolonial animal. J Gerontol A Biol Sci Med Sci 2014;69:1448–61. 24347613 10.1093/gerona/glt201 PMC4271020

[235] Hofmann C, Katus HA, Doroudgar S. Protein misfolding in cardiac disease. Circulation 2019;139:2085–8. 31034286 10.1161/CIRCULATIONAHA.118.037417

[236] Schlossarek S, Englmann DR, Sultan KR, Sauer M, Eschenhagen T, Carrier L. Defective proteolytic systems in Mybpc3-targeted mice with cardiac hypertrophy. Basic Res Cardiol 2012;107:235. 22189562 10.1007/s00395-011-0235-3

[237] Behl C. Breaking BAG: The Co-chaperone BAG3 in health and disease. Trends Pharmacol Sci 2016;37:672–88. 27162137 10.1016/j.tips.2016.04.007

[238] Crum TS, Gleixner AM, Posimo JM, Heat shock protein responses to aging and proteotoxicity in the olfactory bulb. J Neurochem 2015;133:780–94. 25640060 10.1111/jnc.13041 PMC4464935

[239] Gamerdinger M, Hajieva P, Kaya AM, Wolfrum U, Hartl FU, Behl C. Protein quality control during aging involves recruitment of the macroautophagy pathway by BAG3. EMBO J 2009;28:889–901. 19229298 10.1038/emboj.2009.29 PMC2647772

[240] Zhou J, Chong SY, Lim A, Changes in macroautophagy, chaperone-mediated autophagy, and mitochondrial metabolism in murine skeletal and cardiac muscle during aging. Aging 2017;9:583–99. 28238968 10.18632/aging.101181 PMC5361683

[241] Cheng H, Lederer WJ, Cannell MB. Calcium sparks: elementary events underlying excitation-contraction coupling in heart muscle. Science 1993;262:740–4. 8235594 10.1126/science.8235594

[242] Molkentin JD, Lu JR, Antos CL, A calcineurin-dependent transcriptional pathway for cardiac hypertrophy. Cell 1998;93:215–28. 9568714 10.1016/s0092-8674(00)81573-1 PMC4459646

[243] Coppini R, Ferrantini C, Mugelli A, Poggesi C, Cerbai E. Altered Ca^2+^ and Na^+^ homeostasis in human hypertrophic cardiomyopathy: implications for arrhythmogenesis. Front Physiol 2018;9:1391. 30420810 10.3389/fphys.2018.01391 PMC6215954

[244] Louch WE, Bito V, Heinzel FR, Reduced synchrony of Ca^2+^ release with loss of T-tubules-a comparison to Ca^2+^ release in human failing cardiomyocytes. Cardiovasc Res 2004;62:63–73. 15023553 10.1016/j.cardiores.2003.12.031

[245] Gilbert G, Demydenko K, Dries E, Calcium signaling in cardiomyocyte function. Cold Spring Harb Perspect Biol 2020;12:a035428. 31308143 10.1101/cshperspect.a035428 PMC7050587

[246] Kresin N, Stücker S, Krämer E, Analysis of contractile function of permeabilized human hypertrophic cardiomyopathy multicellular heart tissue. Front Physiol 2019;10:239. 30984009 10.3389/fphys.2019.00239 PMC6447666

[247] Song Q, Schmidt AG, Hahn HS, Rescue of cardiomyocyte dysfunction by phospholamban ablation does not prevent ventricular failure in genetic hypertrophy. J Clin Invest 2003;111:859–67. 12639992 10.1172/JCI16738 PMC153769

[248] Helms AS, Alvarado FJ, Yob J, Genotype-dependent and -independent calcium signaling dysregulation in human hypertrophic cardiomyopathy. Circulation 2016;134:1738–48. 27688314 10.1161/CIRCULATIONAHA.115.020086 PMC5127749

[249] Knöll R Myosin binding protein C: implications for signal-transduction. J Muscle Res Cell Motil 2012;33:31–42. 22173300 10.1007/s10974-011-9281-6 PMC3351598

[250] Fraysse B, Weinberger F, Bardswell SC, Increased myofilament Ca^2+^ sensitivity and diastolic dysfunction as early consequences of *Mybpc3* mutation in heterozygous knock-in mice. J Mol Cell Cardiol 2012;52:1299–307. 22465693 10.1016/j.yjmcc.2012.03.009 PMC3370652

[251] Hamilton S, Terentyev D. Altered intracellular calcium homeostasis and arrhythmogenesis in the aged heart. Int J Mol Sci 2019;20:2386. 31091723 10.3390/ijms20102386 PMC6566636

[252] Cooper LL, Li W, Lu Y, Redox modification of ryanodine receptors by mitochondria-derived reactive oxygen species contributes to aberrant Ca^2+^ handling in ageing rabbit hearts. J Physiol 2013;591:5895–911. 24042501 10.1113/jphysiol.2013.260521 PMC3872760

[253] Feridooni HA, Dibb KM, Howlett SE. How cardiomyocyte excitation, calcium release and contraction become altered with age. J Mol Cell Cardiol 2015;83:62–72. 25498213 10.1016/j.yjmcc.2014.12.004

[254] Cohn R, Thakar K, Lowe A, A contraction stress model of hypertrophic cardiomyopathy due to sarcomere mutations. Stem Cell Reports 2019;12:71–83. 30554920 10.1016/j.stemcr.2018.11.015 PMC6335568

[255] Singh RR, Slater RE, Wang J, Distinct mechanisms for increased cardiac contraction through selective alteration of either myosin or troponin activity. JACC Basic Transl Sci 2022;7:1021–37. 36337919 10.1016/j.jacbts.2022.04.013 PMC9626889

[256] Mearini G, Stimpel D, Geertz B, *Mybpc3* gene therapy for neonatal cardiomyopathy enables long-term disease prevention in mice. Nat Commun 2014;5:5515. 25463264 10.1038/ncomms6515

[257] Dutsch A, Wijnker PJM, Schlossarek S, Phosphomimetic cardiac myosin-binding protein C partially rescues a cardiomyopathy phenotype in murine engineered heart tissue. Sci Rep 2019;9:18152. 31796859 10.1038/s41598-019-54665-2 PMC6890639

[258] Prondzynski M, Krämer E, Laufer SD, Evaluation of *Mybpc3* trans-splicing and gene replacement as therapeutic options in human iPSC-derived cardiomyocytes. Mol Ther Nucleic Acids 2017;7:475–86. 28624223 10.1016/j.omtn.2017.05.008 PMC5458066

[259] Ma H, Marti-Gutierrez N, Park SW, Correction of a pathogenic gene mutation in human embryos. Nature 2017;548:413–9. 28783728 10.1038/nature23305

[260] Ho CY, Olivotto I, Jacoby D, Study Design and Rationale of EXPLORER-HCM: evaluation of mavacamten in adults with symptomatic obstructive hypertrophic cardiomyopathy. Circ Heart Fail 2020;13:e006853. 32498620 10.1161/CIRCHEARTFAILURE.120.006853

[261] Sparrow AJ, Watkins H, Daniels MJ, Redwood C, Robinson P. Mavacamten rescues increased myofilament calcium sensitivity and dysregulation of Ca^2+^ flux caused by thin filament hypertrophic cardiomyopathy mutations. Am J Physiol Heart Circ Physiol 2020;318:H715–22. 32083971 10.1152/ajpheart.00023.2020 PMC7099453

[262] Heitner SB, Jacoby D, Lester SJ, Mavacamten treatment for obstructive hypertrophic cardiomyopathy: a clinical trial. Ann Intern Med 2019;170:741–8. 31035291 10.7326/M18-3016

[263] Zhao J, Li Z, Puri R, Molecular profiling of individual FDA-approved clinical drugs identifies modulators of nonsense-mediated mRNA decay. Mol Ther Nucleic Acids 2022;27:304–18. 35024243 10.1016/j.omtn.2021.12.003 PMC8718828

[264] Foinquinos A, Batkai S, Genschel C, Preclinical development of a miR-132 inhibitor for heart failure treatment. Nat Commun 2020;11:633. 32005803 10.1038/s41467-020-14349-2 PMC6994493

[265] Täubel J, Hauke W, Rump S, Novel antisense therapy targeting microRNA-132 in patients with heart failure: results of a first-in-human Phase 1b randomized, double-blind, placebo-controlled study. Eur Heart J 2021;42:178–88. 33245749 10.1093/eurheartj/ehaa898 PMC7954267

[266] Abplanalp WT, Fischer A, John D, Efficiency and target derepression of anti-miR-92a: results of a first in human study. Nucleic Acid Ther 2020;30:335–45. 32707001 10.1089/nat.2020.0871

[267] Alcendor RR, Gao S, Zhai P, Sirt1 regulates aging and resistance to oxidative stress in the heart. Circ Res 2007;100:1512–21. 17446436 10.1161/01.RES.0000267723.65696.4a

[268] Abdellatif M, Sedej S, Carmona-Gutierrez D, Madeo F, Kroemer G. Autophagy in cardiovascular aging. Circ Res 2018;123:803–24. 30355077 10.1161/CIRCRESAHA.118.312208

[269] Alfaras I, Di Germanio C, Bernier M, Pharmacological strategies to retard cardiovascular aging. Circ Res 2016;118:1626–42. 27174954 10.1161/CIRCRESAHA.116.307475 PMC4894351

[270] Watson CJ, Horgan S, Neary R, Epigenetic Therapy for the treatment of hypertension-induced cardiac hypertrophy and fibrosis. J Cardiovasc Pharmacol Ther 2016;21:127–37. 26130616 10.1177/1074248415591698

[271] Mohamed IA, Krishnamoorthy NT, Nasrallah GK, Da'as SI. The role of cardiac myosin binding protein C3 in hypertrophic cardiomyopathy-progress and novel therapeutic opportunities. J Cell Physiol 2017;232:1650–9. 27731493 10.1002/jcp.25639

[272] Xie HF, Liu YZ, Du R, miR-377 induces senescence in human skin fibroblasts by targeting DNA methyltransferase 1. Cell Death Dis 2017;8:e2663. 28277545 10.1038/cddis.2017.75 PMC5386568

[273] Hua Z, Li D, Wu A, Cao T, Luo S. miR-377 inhibition enhances the survival of trophoblast cells via upregulation of FNDC5 in gestational diabetes mellitus. Open Med 2021;16:464–71. 10.1515/med-2021-0247 PMC8005781 33817324

[274] Bhaumik D, Scott GK, Schokrpur S, MicroRNAs miR-146a/b negatively modulate the senescence-associated inflammatory mediators IL-6 and IL-8. Aging 2009;1:402–11. 20148189 10.18632/aging.100042 PMC2818025

[275] Ong J, Woldhuis RR, Boudewijn IM, Age-related gene and miRNA expression changes in airways of healthy individuals. Sci Rep 2019;9:3765. 30842487 10.1038/s41598-019-39873-0 PMC6403379

[276] Santeford A, Lee AY, Sene A, Loss of Mir146b with aging contributes to inflammation and mitochondrial dysfunction in thioglycollate-elicited peritoneal macrophages. Elife 2021;10:e66703. 34423778 10.7554/eLife.66703 PMC8412946

[277] Pan XX, Cao JM, Cai F, Ruan CC, Wu F, Gao PJ. Loss of miR-146b-3p inhibits perivascular adipocyte browning with cold exposure during aging. Cardiovasc Drugs Ther 2018;32:511–8. 30073586 10.1007/s10557-018-6814-x

[278] Zhang X, Azhar G, Wei JY. The expression of microRNA and microRNA clusters in the aging heart. PLoS One 2012;7:e34688. 22529925 10.1371/journal.pone.0034688 PMC3329493

[279] Castanheira CIGD, Anderson JR, Fang Y, Mouse microRNA signatures in joint ageing and post-traumatic osteoarthritis. Osteoarthr Cartil Open 2021;3:100186. 34977596 10.1016/j.ocarto.2021.100186 PMC8683752

[280] Capri M, Olivieri F, Lanzarini C, Identification of miR-31–5p, miR-141–3p, miR-200c-3p, and GLT1 as human liver aging markers sensitive to donor-recipient age-mismatch in transplants. Aging Cell 2017;16:262–72. 27995756 10.1111/acel.12549 PMC5334540

[281] Aunin E, Broadley D, Ahmed MI, Mardaryev AN, Botchkareva NV. Exploring a role for regulatory miRNAs in wound healing during ageing:involvement of miR-200c in wound repair. Sci Rep 2017;7:3257. 28607463 10.1038/s41598-017-03331-6 PMC5468284

[282] Lu J, Li S, Li X, Declined miR-181a-5p expression is associated with impaired natural killer cell development and function with aging. Aging Cell 2021;20:e13353. 33780118 10.1111/acel.13353 PMC8135006

[283] Kim C, Ye Z, Weyand CM, Goronzy JJ. miR-181a-regulated pathways in T-cell differentiation and aging. Immun Ageing 2021;18:28. 34130717 10.1186/s12979-021-00240-1 PMC8203492

[284] Borja-Gonzalez M, Casas-Martinez JC, McDonagh B, Goljanek-Whysall K. Aging science talks: the role of miR-181a in age-related loss of muscle mass and function. Transl Med Aging 2020;4:81–5. 32835152 10.1016/j.tma.2020.07.001 PMC7341035

[285] Goljanek-Whysall K, Soriano-Arroquia A, McCormick R, Chinda C, McDonagh B. miR-181a regulates p62/SQSTM1, parkin, and protein DJ-1 promoting mitochondrial dynamics in skeletal muscle aging. Aging Cell 2020;19:e13140. 32291905 10.1111/acel.13140 PMC7189996

[286] Huan T, Chen G, Liu C, Age-associated microRNA expression in human peripheral blood is associated with all-cause mortality and age-related traits. Aging Cell 2018;17:e12687. 29044988 10.1111/acel.12687 PMC5770777

[287] Ozorhan U, Tuna BG, Cicekdal MB, Long-term chronic caloric restriction alters miRNA profiles in the brain of ageing mice. Br J Nutr 2022;127:641–52. 33823947 10.1017/S0007114521001239

[288] Mendes-Silva AP, Fujimura PT, Silva JRDC, Brain-enriched MicroRNA-184 is downregulated in older adults with major depressive disorder: a translational study. J Psychiatr Res 2019;111:110–20. 30716647 10.1016/j.jpsychires.2019.01.019

[289] Vischioni C, Bove F, De Chiara M, miRNAs copy number variations repertoire as hallmark indicator of cancer species predisposition. Genes 2022;13:1046. 35741808 10.3390/genes13061046 PMC9223155

[290] Budzinska M, Owczarz M, Pawlik-Pachucka E, Roszkowska-Gancarz M, Slusarczyk P, Puzianowska-Kuznicka M. miR-96, miR-145 and miR-9 expression increases, and IGF-1R and FOXO1 expression decreases in peripheral blood mononuclear cells of aging humans. BMC Geriatr 2016;16:200. 27903254 10.1186/s12877-016-0379-y PMC5131432

[291] Mohammed CP, Rhee H, Phee BK, miR-204 downregulates EphB2 in aging mouse hippocampal neurons. Aging Cell 2016;15:380–8. 26799631 10.1111/acel.12444 PMC4783348

[292] Hoss AG, Labadorf A, Beach TG, Latourelle JC, Myers RH. microRNA Profiles in Parkinson's disease prefrontal cortex. Front Aging Neurosci 2016;8:36. 26973511 10.3389/fnagi.2016.00036 PMC4772525

[293] Hoss AG, Labadorf A, Latourelle JC, miR-10b-5p expression in Huntington's disease brain relates to age of onset and the extent of striatal involvement. BMC Med Genomics 2015;8:10. 25889241 10.1186/s12920-015-0083-3 PMC4349621

[294] Nidadavolu LS, Niedernhofer LJ, Khan SA. Identification of microRNAs dysregulated in cellular senescence driven by endogenous genotoxic stress. Aging 2013;5:460–73. 23852002 10.18632/aging.100571 PMC3824412

[295] Ipson BR, Fletcher MB, Espinoza SE, Fisher AL. Identifying exosome-derived microRNAs as candidate biomarkers of frailty. J Frailty Aging 2018;7:100–3. 29741193 10.14283/jfa.2017.45 PMC6384524

[296] Zhang C, Zhang H, Zhao L, Wei Z, Lai Y, Ma X. Differential expression of microRNAs in hypertrophied myocardium and their relationship to late gadolinium enhancement, left ventricular hypertrophy and remodeling in hypertrophic cardiomyopathy. Diagnostics 2022;12:1978. 36010328 10.3390/diagnostics12081978 PMC9406969

[297] Shi H, Li J, Song Q, Systematic identification and analysis of dysregulated miRNA and transcription factor feed-forward loops in hypertrophic cardiomyopathy. J Cell Mol Med 2019;23:306–16. 30338905 10.1111/jcmm.13928 PMC6307764

[298] Derda AA, Thum S, Lorenzen JM, Blood-based microRNA signatures differentiate various forms of cardiac hypertrophy. Int J Cardiol 2015;196:115–22. 26086795 10.1016/j.ijcard.2015.05.185 PMC4936391

[299] Roncarati R, Viviani Anselmi C, Losi MA, Circulating miR-29a, among other up-regulated microRNAs, is the only biomarker for both hypertrophy and fibrosis in patients with hypertrophic cardiomyopathy. J Am Coll Cardiol 2014;63:920–7. 24161319 10.1016/j.jacc.2013.09.041

[300] Rusu-Nastase EG, Lupan AM, Marinescu CI, Neculachi CA, Preda MB, Burlacu A. MiR-29a Increase in aging may function as a compensatory mechanism against cardiac fibrosis through SERPINH1 downregulation. Front Cardiovasc Med 2021;8:810241. 35118144 10.3389/fcvm.2021.810241 PMC8804242

[301] Heid J, Cencioni C, Ripa R, Age-dependent increase of oxidative stress regulates microRNA-29 family preserving cardiac health. Sci Rep 2017;7:16839. 29203887 10.1038/s41598-017-16829-w PMC5715159

[302] Li M, Chen X, Chen L, Chen K, Zhou J, Song J. MiR-1–3p that correlates with left ventricular function of HCM can serve as a potential target and differentiate HCM from DCM. J Transl Med 2018;16:161. 29885652 10.1186/s12967-018-1534-3 PMC5994246

[303] Sun TY, Li YQ, Zhao FQ, MiR-1–3p and MiR-124–3p synergistically damage the intestinal barrier in the ageing colon. J Crohns Colitis 2022;16:656–67. 34628497 10.1093/ecco-jcc/jjab179 PMC9089420

[304] Hitomi N, Kubo T, Kitaoka H, A frameshift deletion mutation in the cardiac myosin-binding protein C gene associated with dilated phase of hypertrophic cardiomyopathy and dilated cardiomyopathy. J Cardiol 2010;56:189–96. 20605413 10.1016/j.jjcc.2010.04.003

[305] Konno T, Shimizu M, Ino H, A novel missense mutation in the myosin binding protein-C gene is responsible for hypertrophic cardiomyopathy with left ventricular dysfunction and dilation in elderly patients. J Am Coll Cardiol 2003;41:781–6. 12628722 10.1016/s0735-1097(02)02957-1

[306] Yang QL, Zuo L, Ma ZL, Gender- and age-related differences in distinct phenotypes of hypertrophic cardiomyopathy-associated mutation *MYBPC3*-E334K. Heart Vessels 2021;36:1525–35. 33830315 10.1007/s00380-021-01834-x

[307] Sabater-Molina M, Saura D, García-Molina Sáez E, A novel founder mutation in MYBPC3: phenotypic comparison with the most prevalent MYBPC3 mutation in Spain. Rev Esp Cardiol 2017;70:105–14. 28029522 10.1016/j.rec.2016.06.020

[308] Kang C, Xu Q, Martin TD, The DNA damage response induces inflammation and senescence by inhibiting autophagy of GATA4. Science 2015;349:aaa5612. 26404840 10.1126/science.aaa5612 PMC4942138

[309] Jiao H, Walczak BE, Lee MS, Lemieux ME, Li WJ. GATA6 regulates aging of human mesenchymal stem/stromal cells. Stem Cells 2021;39:62–77. 33252174 10.1002/stem.3297 PMC7772271

[310] Deczkowska A, Matcovitch-Natan O, Tsitsou-Kampeli A, Mef2C restrains microglial inflammatory response and is lost in brain ageing in an IFN-I-dependent manner. Nat Commun 2017;8:717. 28959042 10.1038/s41467-017-00769-0 PMC5620041

[311] Rogers NH, Landa A, Park S, Smith RG. Aging leads to a programmed loss of brown adipocytes in murine subcutaneous white adipose tissue. Aging Cell 2012;11:1074–83. 23020201 10.1111/acel.12010 PMC3839316

[312] Hsieh PN, Sweet DR, Fan L, Jain MK. Aging and the Krüppel-like factors. Trends Cell Mol Biol 2017;12:1–15. 29416266 PMC5798252

[313] Sheydina A, Volkova M, Jiang L, Linkage of cardiac gene expression profiles and ETS2 with lifespan variability in rats. Aging Cell 2012;11:350–9. 22247964 10.1111/j.1474-9726.2012.00794.x PMC3306452

[314] Ma X, Warnier M, Raynard C, The nuclear receptor RXRA controls cellular senescence by regulating calcium signaling. Aging Cell 2018;17:e12831. 30216632 10.1111/acel.12831 PMC6260923

[315] Martin N, Ma X, Bernard D. Regulation of cellular senescence by retinoid X receptors and their partners. Mech Ageing Dev 2019;183:111131. 31476329 10.1016/j.mad.2019.111131

[316] Natrajan MS, de la Fuente AG, Crawford AH, Retinoid X receptor activation reverses age-related deficiencies in myelin debris phagocytosis and remyelination. Brain 2015;138:3581–97. 26463675 10.1093/brain/awv289 PMC4668920

[317] Grifone R, Shao M, Saquet A, Shi DL. RNA-Binding protein Rbm24 as a multifaceted post-transcriptional regulator of embryonic lineage differentiation and cellular homeostasis. Cells 2020;9:1891. 32806768 10.3390/cells9081891 PMC7463526

[318] Tabrez SS, Sharma RD, Jain V, Siddiqui AA, Mukhopadhyay A. Differential alternative splicing coupled to nonsense-mediated decay of mRNA ensures dietary restriction-induced longevity. Nat Commun 2017;8:306. 28824175 10.1038/s41467-017-00370-5 PMC5563511

[319] Matecic M, Smith DL, Pan X, A microarray-based genetic screen for yeast chronological aging factors. PLoS Genet 2010;6:e1000921. 20421943 10.1371/journal.pgen.1000921 PMC2858703

[320] Deka B, Chandra P, Singh KK. Functional roles of human Up-frameshift suppressor 3 (UPF3) proteins: from nonsense-mediated mRNA decay to neurodevelopmental disorders. Biochimie 2021;180:10–22. 33132159 10.1016/j.biochi.2020.10.011

[321] Nixon RA. The calpains in aging and aging-related diseases. Ageing Res Rev 2003;2:407–18. 14522243 10.1016/s1568-1637(03)00029-1

[322] Ong SB, Lee WH, Shao NY, Calpain inhibition restores autophagy and prevents mitochondrial fragmentation in a human iPSC model of diabetic endotheliopathy. Stem Cell Reports 2019;12:597–610. 30799273 10.1016/j.stemcr.2019.01.017 PMC6411483

[323] Thompson J, Maceyka M, Chen Q. Targeting ER stress and calpain activation to reverse age-dependent mitochondrial damage in the heart. Mech Ageing Dev 2020;192:111380. 33045249 10.1016/j.mad.2020.111380 PMC7686231

[324] Altun M, Besche HC, Overkleeft HS, Muscle wasting in aged, sarcopenic rats is associated with enhanced activity of the ubiquitin proteasome pathway. J Biol Chem 2010;285:39597–608. 20940294 10.1074/jbc.M110.129718 PMC3000941

[325] Gumucio JP, Mendias CL. Atrogin-1, MuRF-1, and sarcopenia. Endocrine 2013;43:12–21. 22815045 10.1007/s12020-012-9751-7 PMC3586538

[326] Clavel S, Coldefy AS, Kurkdjian E, Salles J, Margaritis I, Derijard B. Atrophy-related ubiquitin ligases, atrogin-1 and MuRF1 are up-regulated in aged rat tibialis anterior muscle. Mech Ageing Dev 2006;127:794–801. 16949134 10.1016/j.mad.2006.07.005

[327] Whitman SA, Wacker MJ, Richmond SR, Godard MP. Contributions of the ubiquitin-proteasome pathway and apoptosis to human skeletal muscle wasting with age. Pflugers Arch 2005;450:437–46. 15952031 10.1007/s00424-005-1473-8

[328] Raue U, Slivka D, Jemiolo B, Hollon C, Trappe S. Proteolytic gene expression differs at rest and after resistance exercise between young and old women. J Gerontol A Biol Sci Med Sci 2007;62:1407–12. 18166693 10.1093/gerona/62.12.1407

[329] Edström E, Altun M, Hägglund M, Ulfhake B. Atrogin-1/MAFbx and MuRF1 are downregulated in aging-related loss of skeletal muscle. J Gerontol A Biol Sci Med Sci 2006;61:663–74. 16870627 10.1093/gerona/61.7.663

[330] Haddad F, Adams GR. Aging-sensitive cellular and molecular mechanisms associated with skeletal muscle hypertrophy. J Appl Physiol 2006;100:1188–203. 16373446 10.1152/japplphysiol.01227.2005

[331] Mota R, Parry TL, Yates CC, Increasing cardiomyocyte atrogin-1 reduces aging-associated fibrosis and regulates remodeling in vivo. Am J Pathol 2018;188:1676–92. 29758183 10.1016/j.ajpath.2018.04.007 PMC6026801

[332] Zaglia T, Milan G, Ruhs A, Atrogin-1 deficiency promotes cardiomyopathy and premature death via impaired autophagy. J Clin Invest 2014;124:2410–24. 24789905 10.1172/JCI66339 PMC4038560

[333] Wang F, He Q, Gao Z, Redington AN. Atg5 knockdown induces age-dependent cardiomyopathy which can be rescued by repeated remote ischemic conditioning. Basic Res Cardiol 2021;116:47. 34319513 10.1007/s00395-021-00888-2 PMC8316897

[334] Hartleben B, Gödel M, Meyer-Schwesinger C, Autophagy influences glomerular disease susceptibility and maintains podocyte homeostasis in aging mice. J Clin Invest 2010;120:1084–96. 20200449 10.1172/JCI39492 PMC2846040

[335] Lipinski MM, Zheng B, Lu T, Genome-wide analysis reveals mechanisms modulating autophagy in normal brain aging and in Alzheimer's disease. Proc Natl Acad Sci USA 2010;107:14164–9. 20660724 10.1073/pnas.1009485107 PMC2922576

[336] Pyo JO, Yoo SM, Ahn HH, Overexpression of Atg5 in mice activates autophagy and extends lifespan. Nat Commun 2013;4:2300. 23939249 10.1038/ncomms3300 PMC3753544

