# Supplementary material for: Hypertrophic cardiomyopathy in MYBPC3 carriers in aging
Source: J Cardiovasc Aging. Author manuscript; Available in PMC 2024 Mar 7. (PMC10883298; doi:10.20517/jca.2023.29)
Supplement: Supplementary Material [file NIHMS1962005-supplement-Supplementary_Material.pdf]

Name of Journal: The Journal of Cardiovascular Aging
Manuscript NO: jca4029
Manuscript Type: Review

Hypertrophic cardiomyopathy in *MYBPC3* carriers in aging

Kalyani Ananthamohan[1], Julian E. Stelzer[2], Sakthivel Sadayappan[1]

[1]Department of Internal Medicine, Division of Cardiovascular Health and Disease, University of Cincinnati, Cincinnati, OH 45267, USA.
[2]Department of Physiology and Biophysics, School of Medicine, Case Western Reserve University, Cleveland, OH 45267, USA.

Correspondence to: Kalyani Ananthamohan, Ph.D., Division of Cardiovascular Health and Disease, University of Cincinnati, 231 Albert Sabin Way, Cincinnati, OH 45267, USA. E-mail: ananthka@ucmail.uc.edu; Sakthivel Sadayappan, Ph.D., MBA, Division of Cardiovascular Health and Disease, University of Cincinnati, 231 Albert Sabin Way, Cincinnati, OH 45267, USA. E-mail: sadayasl@ucmail.uc.edu

ORCID: Kalyani Ananthamohan(0000-0003-4794-9144); Sakthivel Sadayappan(0000-0003-2006-7678)

How to cite this article: Ananthamohan K, Stelzer JE, Sadayappan S. Hypertrophic cardiomyopathy in *MYBPC3* carriers in aging. *J Cardiovasc Aging* 2024;4:xx. http://dx.doi.org/10.20517/jca.2023.29

Abbreviations:

ACM Arrhythmogenic Cardiomyopathy

AF Atrial Fibrillation

Akt protein kinase B

BRG1 *SWI/SNF Related*, *Matrix Associated*, *Actin Dependent Regulator of Chromatin* A4

C. elegans Caenorhabditis elegans

ChIP Chromatin Immunoprecipitation

cMyBP-C Cardiac Myosin Binding Protein-C

DCM Dilated Cardiomyopathy

EAP Epigenetic aging program

ECG Electrocardiogram

ETS2 ETS proto-oncogene 2

GATA GATA binding protein

HCM Hypertrophic Cardiomyopathy

HF Heart Failure

IGF-1 Insulin-like growth factor 1

KLF15 Krüppel-like factor 15

LV Left Ventricle

miRNA microRNA

mTOR Mammalian target of rapamycin

MYBPC3 Cardiac Myosin Binding Protein-C gene

MYH7 β-Myosin Heavy Chain

NMD Nonsense-mediated mRNA decay

PI3K Phosphoinositide 3-kinase

PKA Protein Kinase A

PTC Premature Termination of Codons

RNAi RNA interference

RXRA *Retinoid X receptor alpha* (*RXR-alpha)*

SCD Sudden Cardiac Death

SFPQ Splicing factor proline- and glutamine-rich

SIRT Sirtuin

SMAD Suppressor of Mothers against Decapentaplegic

SMG SMG1 nonsense-mediated mRNA decay associated PI3K-related kinase

SRSF *serine/arginine-rich splicing factors*

TGF β 1 Transforming growth factor beta 1

UPS Ubiquitin-proteosome system

ZBTB16 *Zinc finger and BTB domain-containing protein 16*
